# Statistical and Block Copolymers of n-Dodecyl and Allyl Isocyanate via Titanium-Mediated Coordination Polymerization: A Route to Polyisocyanates with Improved Thermal Stability

**DOI:** 10.3390/polym16243537

**Published:** 2024-12-19

**Authors:** Maria Iatrou, Aikaterini Katara, Panagiotis A. Klonos, Apostolos Kyritsis, Marinos Pitsikalis

**Affiliations:** 1 Industrial Chemistry Laboratory, Department of Chemistry, National and Kapodistrian University of Athens, Panepistimiopolis Zografou, 15771 Athens, Greece; iatmaria97@gmail.com (M.I.); katerinakatara01@gmail.com (A.K.); 2 Dielectrics Group, Physics Department, School of Applied Mathematical and Physics Science, National Technical University of Athens, 9 Heroon Polytechniou, 15780 Zografou, Greece; panos48al@gmail.com (P.A.K.); akyrits@central.ntua.gr (A.K.)

**Keywords:** n-dodecyl isocyanate, allyl isocyanate, coordination polymerization, half-titanocene complex, statistical copolymers, block copolymers, thermal degradation, thiol-ene click reaction

## Abstract

Well-defined amorphous/semi-crystalline statistical copolymers of n-dodecyl isocyanate, DDIC, and allyl isocyanate, ALIC, were synthesized via coordination polymerization using the chiral half-titanocene complex CpTiCl_2_(O-(S)-2-Bu) as an initiator. In the frame of the terminal model, the monomer reactivity ratios of the statistical copolymers were calculated using both well-known linear graphical methods and the computer program COPOINT. The molecular and structural characteristics of the copolymers were also calculated. The thermal properties of these samples were studied by differential scanning calorimetry, DSC, measurements. The kinetics of the thermal decomposition of the statistical copolymers was studied by thermogravimetric analysis, TGA, and differential thermogravimetry, DTG, and the activation energy of this process was calculated by employing several theoretical models. Moreover, block copolymers with the structure P[DDIC-b-(DDIC-co-ALIC)] were synthesized by sequential addition of monomers and coordination polymerization methodologies. The samples were characterized by nuclear magnetic resonance, NMR, spectroscopy; size exclusion chromatography, SEC; and DSC. The thermal stability of the blocks was also studied by TGA and DTG and compared to the corresponding statistical copolymers, showing that the macromolecular architecture greatly affects the properties of the copolymers. A thiol-ene click post-polymerization reaction was performed to introduce aromatic groups along the polyisocyanate chain in order to improve the thermal stability of the parent polymers. Evidently, these statistical and block copolymers can be employed as precursors for the synthesis of novel polyisocyanate-based materials.

## 1. Introduction

Coordination polymerization techniques have emerged through the years as a valuable tool for the synthesis of novel polymeric materials [1]. Ziegler and Natta initially employed their two-component catalytic systems to promote the stereospecific catalytic polymerization of α-olefins [2,3]. Kaminsky and coworkers efficiently activated metallocene complexes using suitable cocatalysts for the polymerization of α-olefins [4,5]. These catalytic systems were later successfully employed for the polymerization of polar monomers, such as methacrylates and acrylates [6,7]. Since the activated metallocenes are cationically charged, they were also employed as cationic initiators for the polymerization of isobutylene, lactones, oxazolines, and vinyl ethers [8,9,10], revealing that the same system could be applied both as a catalyst and/or as an initiator [11]. In addition, ring opening metathesis polymerization reactions were realized using the well-defined alkylidene Grubbs and Schrock catalysts based on Ruthenium and Molybdenum, respectively, or other more exotic transition metal complexes, leading to the polymerization of strained cyclic olefins, such as cyclopentene, cyclooctene, norbornene and its derivatives, etc. [12,13]. Non-metallocene along with late transition metal complexes have been also prepared and tested for the polymerization of non-polar and polar monomers [14,15].

Recent advances in coordination chemistry have allowed for the synthesis of a variety of transition metal complexes, offering control over the molecular characteristics and the stereochemistry of the produced polymers [16,17], as well as allowing for the synthesis of well-defined complex macromolecular architectures [18,19].

A special application of coordination polymerization refers to the family of isocyanate, IC, monomers [20]. Novak was the first to report the employment of half-titanocene complexes bearing an alkoxide ligand as initiators for the controlled polymerization of ICs [21,22,23]. Up to that time, anionic polymerization was the only tool to polymerize ICs [24]. The major problem with this polymerization technique was the presence of back-biting reactions, leading to the formation of thermodynamically stable cyclotrimers and, therefore, to the depolymerization procedure, making it difficult to synthesize polymers with predetermined molecular weights and narrow molecular weight distributions [25,26]. Even after the development of more efficient anionic initiators and additives that fortify the controlled/living character of the polymerization reaction, the situation was not appreciably improved [27,28,29,30,31,32,33,34,35,36,37].

The coordination polymerization of ICs offered the possibility to avoid back-biting reactions and to perform the procedure at room temperature. Moreover, it was possible to synthesize end-functionalized polymers and well-defined complex macromolecular architectures, such as block copolymers, graft copolymers, block–graft copolymers, star homopolymers, and miktoarm star copolymers [38,39,40,41,42,43,44,45,46,47,48,49]. The major drawback of this synthetic approach towards the polymerization of ICs is that the propagation step is an equilibrium reaction. Therefore, in order to achieve high yields and lead to high-molecular-weight products, it is imperative to work in highly concentrated solutions. Under these conditions, the solution’s viscosity remains very high, and therefore, it is difficult to control the polymerization rate, leading to products with increased polydispersities. A solution to avoid this complication is to terminate the polymerization at lower yields before the substantial increase in the viscosity of the polymerization medium.

All these efforts to control the polymerization of ICs are due to the high impact the corresponding polymers have on theoretical investigations and practical applications. The polyisocyanates, PICs, adopt a stiff dynamic helical configuration both in solution and in the solid state [50,51,52,53]. This is the result of the restrictions inhibiting the free rotation around the carbon–nitrogen bond of the amide linkage due to the resonance effect with the carbonyl group of the amide moiety and the steric hindrance introduced from the side group, which is linked to the main macromolecular backbone. Consequently, PICs have interesting liquid crystalline properties and can be used in several applications, such as optical switches, photonic crystals, nanocarriers, and, more recently, in biomimetics, since they resemble the polyamide structure of proteins, and in addition, they can be beneficial to understand the biological function of other biomolecules, such as DNA and RNA [54,55,56,57,58,59,60,61,62].

In this work, the synthesis of statistical and block copolymers of allyl, ALIC, and n-dodecyl isocyanate, DDIC, is described. The polymerization of ALIC has been reported to take place both via anionic [63,64] and coordination polymerization [65]. Solubility problems have been reported for poly(allyl isocyanate), PALIC, employing anionic polymerization methodologies, probably due to possible crosslinking reactions involving the pendant allyl group of the monomer. Similar problems have not been reported for the synthesis of PALIC through coordination polymerization chemistry, even for molecular weights as high as 10^6^, meaning that the side allyl group remains intact during the reaction. The copolymerization of n-hexyl isocyanate, HIC, and ALIC has been reported in the literature, employing 2,2,2-trifluoroethoxy titanium trichloride, TiCl_3_(OCH_2_CH_3_) as initiator in toluene solutions at room temperature [65]. In the present study, the complex CpTiCl_2_(O-(S)-2-Bu), derived from CpTiCl_3_ and (S)-2-butanol, was used as the initiator. The replacement of one of the chlorine atoms of titanium from its coordination sphere with the cyclopentadienyl, Cp, group reduces the Lewis acidity of the metal due to the presence of the stereochemically hindered and electron-donating Cp moiety. This change usually leads to lower rates of polymerization, but in a more controlled manner.

The copolymers were characterized by size exclusion chromatography, SEC, and nuclear magnetic resonance, NMR, spectroscopy. A post-polymerization reaction was employed to increase the thermal stability of the copolymers. The thermal properties of the samples were studied by differential scanning calorimetry, DSC, whereas the thermal stability and the kinetics of the thermal decomposition were studied by thermogravimetric analysis, TGA, and differential thermogravimetry, DTG.

## 2. Materials and Methods

### 2.1. Materials

The complex [CpTiCl_2_(O-(S)-2-Bu)] was synthesized according to previously reported protocols [43] and used as initiator for all the polymerizations. CpTiCl_3_ (Aldrich Europe, Buchs, Switzerland, 97%) and (S)-2-butanol (Aldrich, 99%, St. Louis, MI, USA) were used as received for the preparation of the initiator, while triethylamine (Aldrich, 98%) and chloroform-d were dried over CaH_2_ and molecular sieves (Linde 4Å, Doublin, Ireland) for 24 h, respectively, and then both distilled under reduced pressure. Toluene (Fisher, ≥99.8%, Hampton, NH, USA) was dried over CaH_2_ overnight and then distilled. Dichloromethane (Fisher, ≥99.8%), used for the thiol-ene click reaction, was dried over molecular sieves for 20–24 h and distilled. Azobisisobutyronitrile, AIBN (98% ACROS), was purified by recrystallization twice from methanol and then vacuum-dried. Methanol (Aldrich, 98%) and 2-Phenylethanethiol (TCI, Tokyo Japan, >97%) were used as received. For the purification of allyl isocyanate, ALIC (Sigma-Aldrich, 98%, St. Louis, MI, USA), and dodecyl isocyanate, DDIC (Sigma-Aldrich, 99%), the two monomers were left under stirring for 24 h with a small amount of drying CaH_2_ as well as 4,4-methylene-bis-phenyl isocyanate, followed by distillation.

All the purification and synthetic procedures were performed using a combination of Schlenk or glove box techniques [66,67], unless stated differently.

### 2.2. Synthesis of Homopolymer PDDIC

In a 10 mL vial, the required amount of initiator (0.024 g, 0.09 mmol) was added and dissolved in 0.5 mL toluene under an inert atmosphere at room temperature. DDIC (1.0 mL, 4.12 mmol) was then added to the yellow solution, leading to the formation of an orange solution with increased viscosity after 3 h. After 4 h, the vial was removed from the inert atmosphere chamber and placed in an ice bath, and the solution was diluted with approximately 3 mL of dichloromethane. The termination of the polymerization was carried out by adding 0.5 mL of methanol under stirring. The solution was transferred dropwise to a beaker containing 100 mL of ice-cold methanol under stirring, and a white solid precipitated. Following filtration through a Por.4 sintered glass funnel, the product was dried under vacuum for 72 h, and the yield was determined gravimetrically equal to 40.6%.

### 2.3. Synthesis of Homopolymer PALIC and Kinetic Study

A typical procedure for the homopolymerization of ALIC follows the same synthetic route as described for the synthesis of PDDIC. A series of experiments was conducted at various polymerization times and concentrations to determine the optimal conditions for polymerization. These conditions include toluene as the solvent, monomer concentrations higher than 0.015 M, and room temperature for the polymerization reaction. In order to study the kinetics, a series of four experiments was carried out in identical conditions. In particular, four vials containing a magnetic stirring bar, a 0.156 M solution of [CpTiCl_2_(O(S)-2-Bu)] in toluene (0.75 mL), and ALIC (1.0 mL) were stirred at room temperature in an inert atmosphere for 15, 30, 45, and 60 min, respectively. The reaction progress was monitored by ^1^H NMR and SEC experiments.

### 2.4. Synthesis of Statistical Copolymers, P(ALIC-co-DDIC)

In order to determine the reactivity ratios of the monomers, five distinct copolymerization experiments were conducted under identical conditions, with a different molar feed ratio of the two monomers (ALIC/DDIC: 20/80, 40/60, 50/50, 60/40, 80/20) in each instance. The methodology employed was consistent for all five experiments. Under the inert atmosphere, the necessary quantity of the complex was transferred to a 10 mL dry vial and dissolved in toluene. The appropriate amount of monomers (ALIC and DDIC) was then added simultaneously and left under stirring for several hours at room temperature. In each case, the polymerization was terminated when the viscosity was increased by removing the vial from the inert atmosphere chamber, placing it into an ice bath, and adding a frozen mixture of MeOH/CH_2_Cl_2_ at a 1/10 ratio. Subsequently, the solid product was precipitated in methanol and filtered through a Por. 4 sintered glass funnel in a Buchner apparatus under vacuum. Finally, the solid was dried under reduced pressure for 72 h and the yield was determined gravimetrically (lower than 50% in all cases).

### 2.5. Synthesis of Block Copolymers, P[DDIC-b-(DDIC-co-ALIC)]

A series of three block copolymers of DDIC and ALIC were prepared in different feed ratios (monomer molar ratios DDIC/ALIC: 20/80, 50/50, 80/20). In each case, the polymerization of DDIC was initially performed followed by the polymerization of the ALIC monomer. In a typical process, the following compounds were added sequentially to a glass vial: [CpTiCl_2_(-O-(s)-2-Bu)], toluene, and DDIC. The yellow transparent solution was left under stirring at room temperature under an inert atmosphere. As the polymerization progressed, the solution exhibited a turbid orange color and a notable increase in viscosity. Following a reaction period during which the mixture became very viscous, ALIC was added along with toluene to facilitate the stirring process and ensure the homogeneous incorporation of the second monomer. After several hours, when the solution exhibited a dramatic increase in viscosity again, the reaction mixture was diluted with dichloromethane, cooled to 0 °C, and methanol was added to terminate the polymerization process. Subsequently, the solid product was precipitated by the dropwise addition of the solution to a beaker containing 150 mL of cold methanol under stirring, thereby forming a white solid, which was then filtered and dried as described above.

### 2.6. Synthesis of P3PETPIC and P(3PETPIC-co-DDIC)

Poly(3-(phenylthio)-propyl isocyanate), P3PETPIC, was prepared by the following procedure. In a 50 mL Schlenk flask containing a magnetic stirring bar, PALIC (0.15 g) was dissolved in dichloromethane (3 mL). Afterwards, AIBN (0.015 g, 0.09 mmol) and 2-phenylethanethiol (0.29 mL, 2.17 mmol) were added in the solution under continuous argon flow. The solution was degassed and the flask was immersed in a preheated oil bath at 50 °C. Following a period of 24 h, the solution remained under stirring, yet exhibited increased viscosity. The content of the Schlenk flask was then diluted in dichloromethane and transferred to a 250 mL beaker containing methanol (100 mL). The white solid was filtered through a Por.4 sintered glass funnel and was dried under vacuum for 24 h. The final product displayed a viscoelastic texture.

In regard to the synthesis of the [ P(3PETPIC-co-DDIC)], the following reagents were sequentially added to a 50 mL Schlenk flask under a continuous flow of inert gas: 0.2 g P(ALIC-co-DDIC) 60/40, 3 mL dichloromethane, AIBN (0.012 g, 0.073 mmol), and 2-phenylethanethiol (0.23 mL, 2.25 mmol). The reaction was carried out under identical conditions with the synthesis of P3PETPIC, and the solid product was received following the same procedure as previously described.

### 2.7. Characterization

The molecular weights (M_w_ and M_n_) and molecular weight distribution, Ð, were determined by size exclusion chromatography (SEC) using a modular instrument comprising a Waters (Milford, MA, USA) model 600 pump, a Waters 2690 automated sample injector, a Waters 2414 refractive index detector, and 4μ-Styragel columns with a continuous porosity range from 500 to 10^6^ Å. Eight polystyrene standards with molecular weights in the range of 2500–600,000 were used for the calibration, while the carrier solvent was chloroform, with the flow rate set at 1.0 mL/min. The NMR measurements were carried out on a Bruker Avance Neo 400 MHz spectrometer using chloroform-d as a solvent at 25 °C. Thermogravimetric analysis (TGA) and differential thermogravimetry (DTG) were employed to study the thermal stability and the kinetics of thermal decomposition of the copolymers and the respective homopolymers. This was conducted utilizing a Q50 TGA model from TA Instruments, with the samples being heated from ambient temperatures to 600 °C in a 40 mL/min flow of nitrogen at heating rates of 3, 5, 7, 10, 15, and 20 °C/min. The glass transition temperatures were determined by differential scanning calorimetry (DSC) using a Q200 DSC model from TA Instruments (New Castle, DE, USA). The samples were heated under a nitrogen atmosphere at a rate of 10 °C/min from −100 °C to 100 °C. Results from the second heating and second cooling cycles were obtained in all cases.

## 3. Results and Discussion

### 3.1. Homopolymerization of ALIC and Kinetic Study

**Scheme 1:**
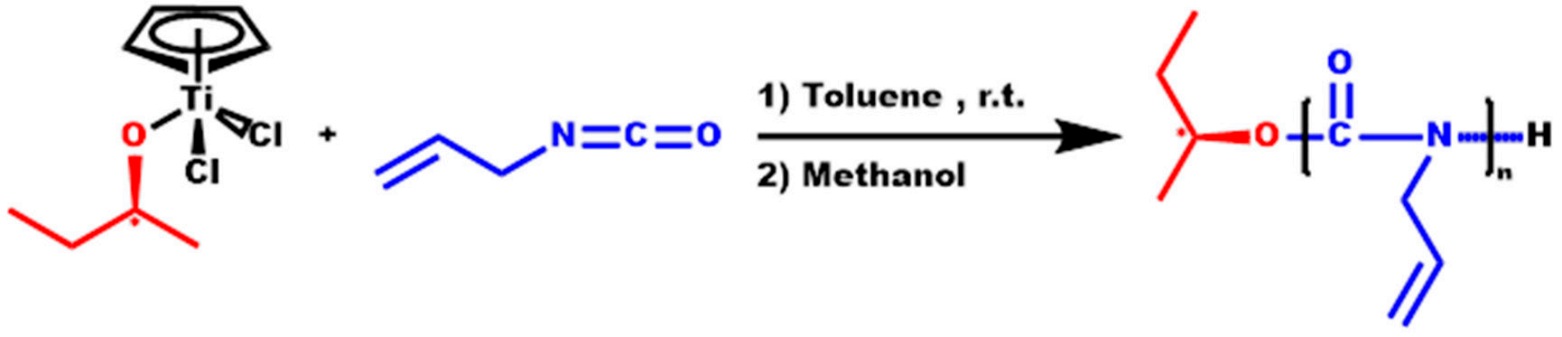
Synthesis of PALIC.

A series of homopolymerizations were conducted in order to optimize the experimental conditions of the reaction and generate the highest possible yield while maintaining a narrow molecular weight distribution (Scheme 1). The best results were obtained keeping the polymerization conversion at relatively low values (less than 50%) and conducting the polymerization reaction at high concentrations. Given that the polymerization of ICs via organotitanium initiation is fully reversible between the polymer and monomer, the concentration of the monomer is a significant factor for polymerizing isocyanates, since the rate of depolymerization is favored over the rate of polymerization in dilute solutions [21,22,23]. In these highly concentrated solutions, the viscosity increases rapidly, leading progressively to a retardation of the rate of polymerization and to phenomena associated with heterogenous polymerization reactions. The most important effect is the broadening of the molecular weight distribution. Therefore, in order to keep low dispersity values, the polymerization yield should be kept at relatively low values (lower than 50% for all samples).

**Figure 1:**
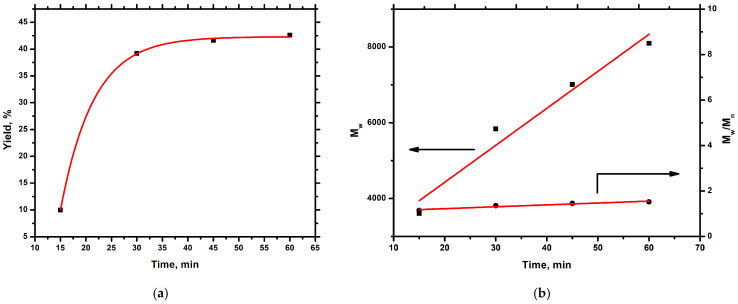
(**a**) Yield vs. time for the homopolymerization of ALIC. (**b**) M_w_ vs. time and M_w_/M_n_ vs. time for the homopolymerization of ALIC.

In order to gain more insight into the polymerization process of ALIC in the presence of the complex [CpTiCl₂(O-(S)-2-Bu)], a kinetic study was carried out. Table 1 reports the results of this study, whereas Figure 1a,b present the time evolution of the polymerization yield, the molecular weight, and the molecular weight distribution. From Figure 1a, it is obvious that the polymerization yield increased rapidly at the initial stages of the polymerization, followed by a retardation phase, due to the increase in the solution viscosity. On the other hand, a linear correlation between the weight average molecular weight, M_w_, and time is evident from Figure 1b, showing that there was considerable control over the polymerization reaction and that termination reactions did not prevail at this stage of the procedure. The molecular weight distribution (M_w_/M_n_) values demonstrate a small but considerable increase over time, again due to the retardation of the polymerization reaction with time (Figure 1b). The SEC traces of the samples prepared from this kinetic analysis are displayed in the Supporting Information Section (SIS) (Figure S1). Samples isolated during the polymerization reaction remained soluble in common organic solvents, and the NMR analysis revealed that the pendant double bonds remained intact with time, meaning that crosslinking did not take place. Therefore, it can be concluded that good control of molecular characteristics is only achieved at relatively low yields.

### 3.2. Homopolymerization of DDIC

**Scheme 2:**
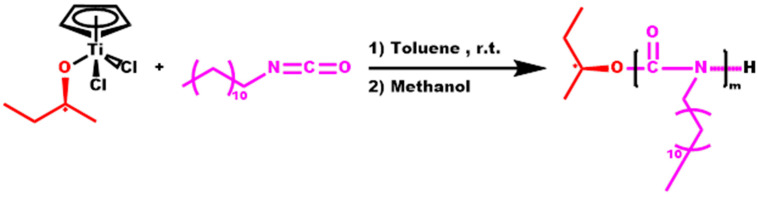
Synthesis of PDDIC.

Poly(dodecyl isocyanate), PDDIC, was successfully synthesized at a low molecular weight (M_w_ = 6 × 10^3^) and a conversion rate of 40.6% (Scheme 2). The distribution was considerably narrow (Ð = 1.16), taking into account previous data related to half-titanocene polymerization systems. The SEC trace of this sample is given in the SIS (Figure S2).

### 3.3. Statistical Copolymers, P(ALIC-co-DDIC)

**Scheme 3:**
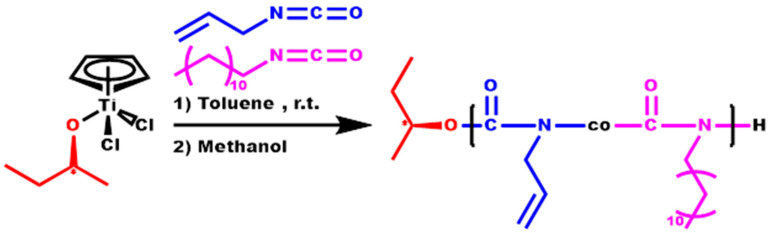
Synthesis of the statistical copolymers P(ALIC-co-PDDIC).

**Figure 2:**
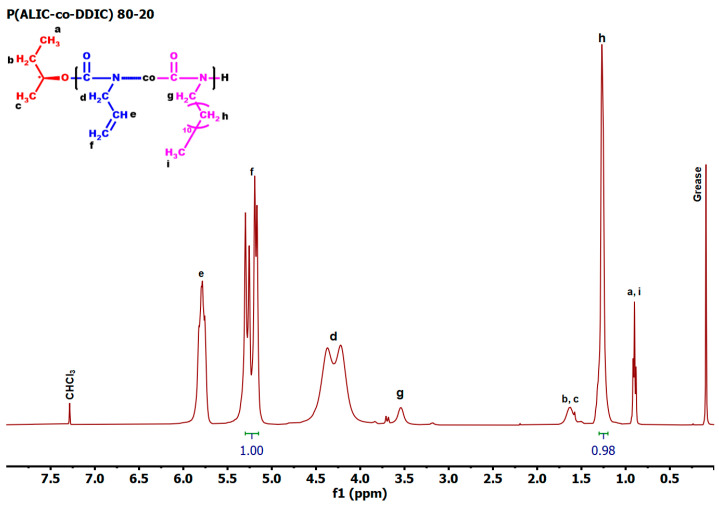
^1^H NMR spectrum of the sample P(ALIC–co–DDIC) 80/20 in CDCl_3_.

**Figure 3:**
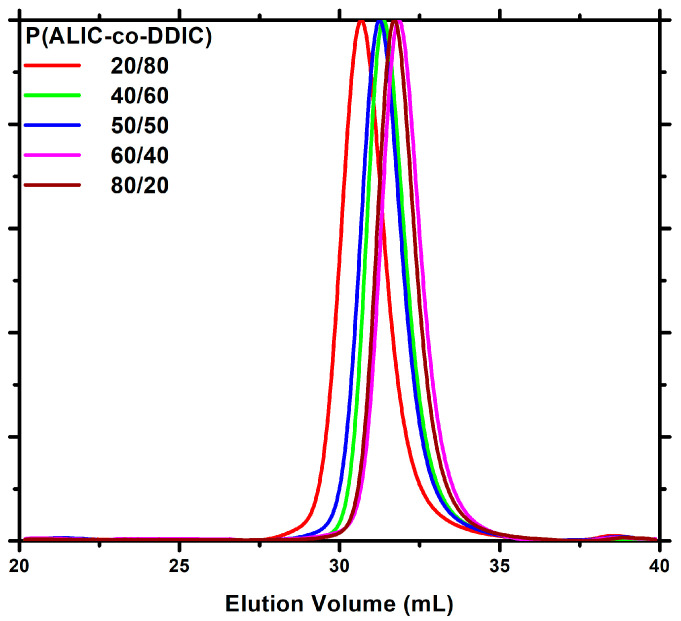
SEC chromatograms of all the P(ALIC–co–DDIC) samples.

Using the complex [CpTiCl_2_(O-(S)-2-Bu)] as the initiator, the copolymerization of ALIC with DDIC was carried out at room temperature (Scheme 3). Five statistical copolymers were successfully synthesized employing different monomer molar ratios of ALIC/DDIC in feed (80/20, 60/40, 50/50, 40/60, and 20/80) in order to calculate the monomer reactivity ratios r_ALIC_ and r_DDIC_. The samples are labeled by numbers, indicating the molar feed ratio of the ALIC and DDIC monomers. The polymeric nature of the products as well as the incorporation of both monomers into the polymer chains were confirmed by ^1^H-NMR spectroscopy and size exclusion chromatography (SEC). The molecular composition was determined by integrating a sharp peak at 1.32 ppm, which was assigned to the methylene groups of the DDIC monomeric units, as well as the range of peaks at 5.0–5.5 ppm, which were attributed to the vinyl protons in the ALIC monomeric units (Figure 2). Low to moderate conversions were intentionally achieved in order to obey the copolymerization equation and, thus, to be able to apply the linear methods for the calculation of the monomer reactivity ratios [68], as will be described in detail below. The SEC traces of the statistical copolymers, illustrated in Figure 3, indicate the presence of symmetrical peaks exhibiting notably low dispersity values. This outcome was achieved due to the relatively low conversions of the copolymerization reactions, thereby confirming that, under the specified experimental conditions, the copolymerization process was effectively controlled. The molecular characteristics of the copolymers are given in Table 2.

### 3.4. Monomer Reactivity Ratios and Statistical Analysis of the Copolymers

The ALIC and DDIC reactivity ratios were determined using the well documented Fineman–Ross (F–R) [69], inverted Fineman–Ross (inv. F–R) [69], Kelen–Tüdos (K–T) [70], and extended Kelen–Tüdos (ext. K–T) [70] linear methods, along with the computer program COPOINT [71]. Further data concerning the established graphical methods can be found in the SIS.

Among the various linear methodologies, the K-T and ext. K-T methods offer the most accurate reactivity ratio values. Nevertheless, it should be noted that all linear methods are constrained by statistical limitations, since the experimental data are assumed to be linear, which is not always the case. In order to overcome these limitations, it is possible to employ a nonlinear approach, such as the COPOINT computer program. COPOINT is a user-friendly software program that numerically integrates the differential copolymerization equations provided by the user and fits them to the experimental composition data. It specifically assesses the copolymerization parameters based on the monomer feed ratio and the copolymer composition data obtained from copolymerization experiments. Additionally, the program provides the user with an estimated error range for the calculated parameters and can be implemented for full monomer conversion.

**Figure 4:**
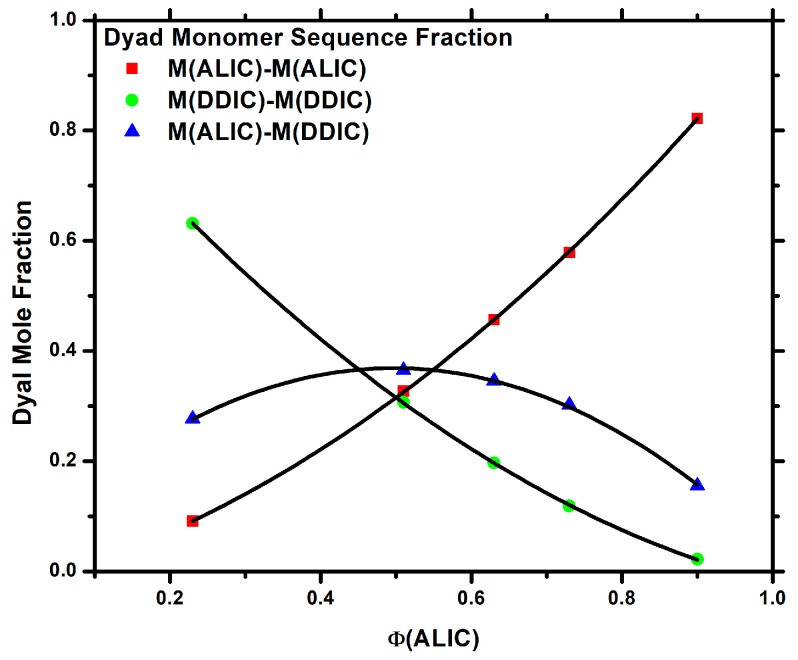
Dyad sequence distribution of the P(ALIC–co–DDIC) copolymers.

The reactivity ratios were calculated taking into account the terminal model, according to which the propagation reaction was exclusively determined by the chemical nature of the monomer, which was the terminal unit of the developing polymer chain [72]. The different methodologies yielded comparable results, as evidenced in Table 3. The plots of the respective methodologies, shown in Figures S3–S6 and Table S1 of the SIS, demonstrate a high degree of linearity, confirming the validity of the terminal model. In each instance, the reactivity ratios of both ALIC and DDIC exceeded unity, with the reactivity ratio of ALIC being notably higher than that of DDIC. The same conclusion was reached by the COPOINT program. This suggests that both monomers in this copolymerization reaction tend to homopolymerize, ultimately forming multiblock copolymers with longer PALIC sequences. This conclusion is also verified by the statistical distribution of the dyad monomer sequences M_ALIC_-M_ALIC_, M_ALIC_-M_DDIC_, and M_DDIC_-M_DDIC_, which was determined using the Igarashi equations [73]. More details related to the calculation method are available in the SIS. Along with the dyad monomer sequences, the mean sequence lengths μ_ALIC_ and μ_DDIC_ were calculated [74], and the results are summarized in Table S2 of the SIS, whereas the variation in the dyad fractions with the ALIC mol fraction in the copolymers is displayed in Figure 4. All the data support the increased tendency of ALIC to homopolymerize compared to DDIC, as well as the formation of multiblock copolymers.

In a previous work, the copolymerization of ALIC with HIC was reported via coordination polymerization using the TiCl_3_(OCH_2_CF_3_) complex [65]. Employing the FR equation, the authors found very different results regarding the reactivity ratios, namely r_ALIC_ = 0.79 and r_HIC_ = 2.05, indicating that HIC has a higher tendency of homopolymerization than ALIC and that the sequences of HIC monomer units along the copolymer chain are much more pronounced than those of ALIC. Therefore, despite the similarities in the two systems, the copolymer chain obtained was distinctively different with the two procedures adopted. The complex CpTiCl_2_(O-(S)-2-Bu) that was employed in the current study led to lower polymerization rates and, thus, better control over the copolymerization reaction. In addition, DDIC, due to the long side alkyl chain, was expected to polymerize in a much lower rate than HIC. Both events seem to favor the polymerization of ALIC over DDIC, leading, finally, to a multiblock structure with longer sequences of ALIC monomer units.

### 3.5. Block Copolymers, P[DDIC-b-(DDIC-co-ALIC)]

**Scheme 4:**
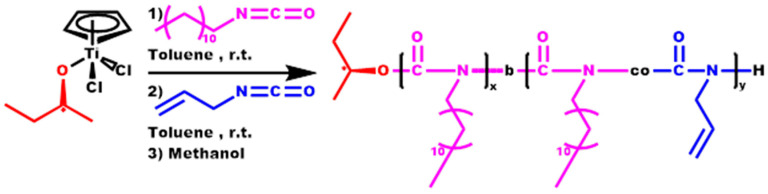
Synthesis of the block copolymers P[DDIC-b-(DDIC-co-ALIC)].

Three block copolymers, each comprising a distinct composition of DDIC and ALIC, were synthesized through the sequential addition of the two monomers into the initiator solution. The second monomer was introduced when the polymerization mixture exhibited a high viscosity, indicating that the synthesis of the first block had reached a sufficient degree of conversion, typically up to 50%. The order of monomer addition was maintained the same in all cases, initiating with the polymerization of DDIC and subsequently incorporating and polymerizing ALIC (Scheme 4). The specific order of addition of the monomers was preferred to exclude the possibility of any type of side reaction, including cross-linking of the double bond of ALIC, which would otherwise result in the formation of undefined products with a broad molecular weight distribution.

Using this methodology, the product is a copolymer with a pure PDDIC first block, whereas the second one is a statistical copolymer containing both DDIC and ALIC monomer units, since the polymerization of the second monomer, ALIC, takes place in the presence of the excess DDIC monomer that remained unreacted after the formation of the first block. Therefore, the final structure is better described as a P[DDIC-b-(DDIC-co-ALIC)] block copolymer. In previous publications [75,76,77], the synthesis of block copolymers of HIC with other ICs was described. In these approaches, the polymerization yield of the first block was allowed to reach higher values (more than 80%), and the excess of the monomer was evaporated in the vacuum line before the addition and the polymerization of the second monomer, giving rise to pure block copolymers. In the present work, DDIC is not volatile enough to follow this procedure, and therefore, the excess of the unreacted monomer after the formation of the first block is still in the reacting mixture, causing the formation of a statistical copolymer of DDIC and ALIC as the second block. The molecular characteristics of these structures are given in Table 4.

The ^1^H-NMR spectra were similar to those of the statistical copolymers. A representative example is given in the SIS (Figure S7). The same peaks were observed, and the copolymer composition was determined by integrating the peaks at 1.32 and 5.0–5.5 ppm. In general, the compositions of the samples appeared to be in close agreement with the stoichiometry employed for the copolymer synthesis, yet a slight inclination towards the incorporation of the DDIC monomeric unit was obvious. This phenomenon can be attributed to the fact that the polymerization of DDIC takes place both as the exclusive component of the first block and as a comonomer with ALIC for the formation of the second block. Therefore, DDIC is almost quantitatively incorporated to the final structure, whereas ALIC has a much lower degree of insertion.

**Figure 5:**
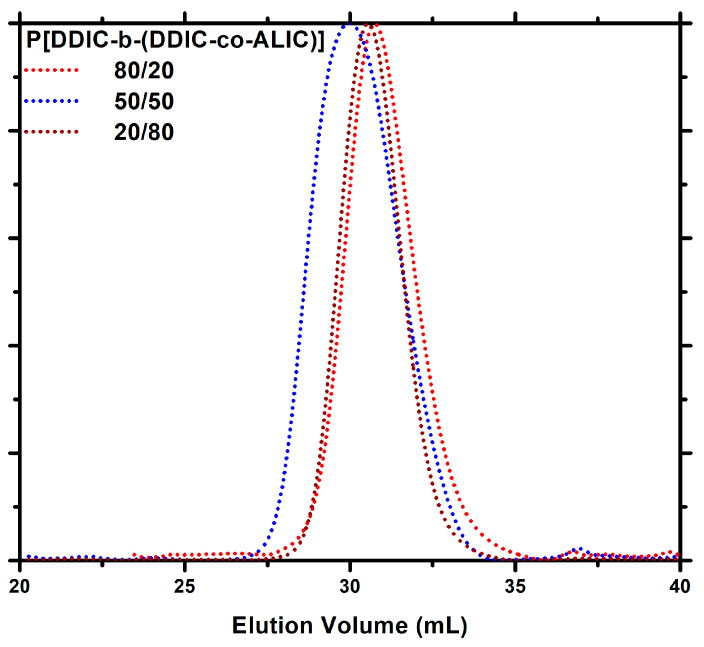
SEC traces of the P[DDIC-b-(DDIC-co-ALIC)] block copolymers.

The synthetic procedure was also monitored by SEC. All the samples possessed remarkably narrow and monomodal distributions, as evidenced by the SEC traces depicted in Figure 5, proving the successful incorporation of the second monomer into the developing chain as well as the effective control of the reaction.

### 3.6. Synthesis of P3PETPIC and P(3PETPIC-co-DDIC) via Thiol-Ene Click Reaction

PICs are thermally sensitive polymers, since their thermal decomposition takes place at relatively low temperature values [75,76,77]. The thermal stability of PICs could be increased by the introduction of side-groups that may retard the thermal degradation. These side groups could be introduced either using specific IC monomers that could be homopolymerized or copolymerized with other more conventional monomers or by performing suitable post-polymerization reaction to preformed PICs. The presence of the allyl side group of the ALIC monomer units offers this possibility. The prerequisite for this procedure is that the functionalization reaction should take place under mild conditions to avoid the thermal decomposition of the parent material or any type of unwanted side reaction, such as crosslinking. In this work, the radical-mediated thiol-ene click reaction was employed [78]. This methodology has previously been applied to PALIC chains to introduce polar hydroxyl groups along the polyisocyanate backbone [63], showing that this procedure is efficient and can be adopted without side effects on the parent chain.

**Scheme 5:**
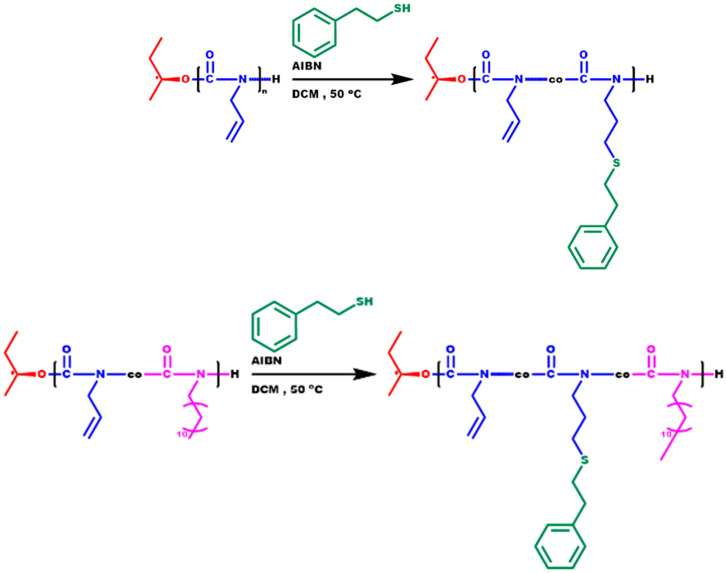
Thiol-ene click reactions.

**Figure 6:**
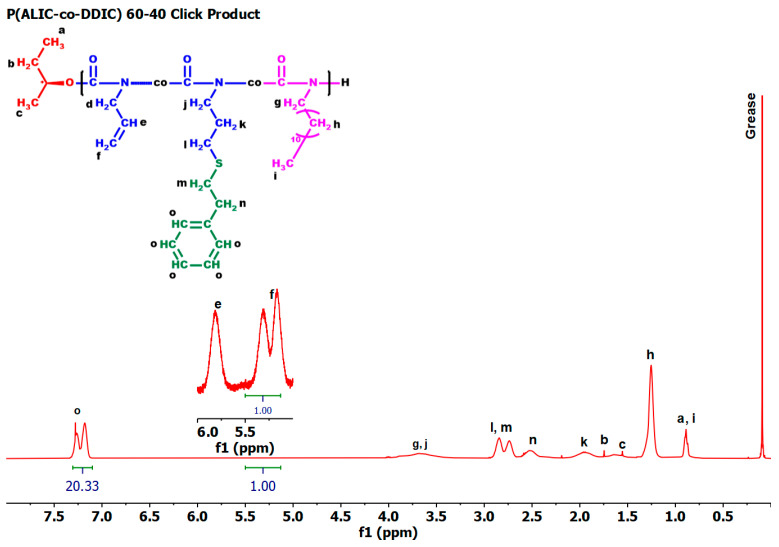
^1^H NMR spectrum of the sample P(ALIC–co–DDIC) 60/40 click product in CDCl_3_.

**Figure 7:**
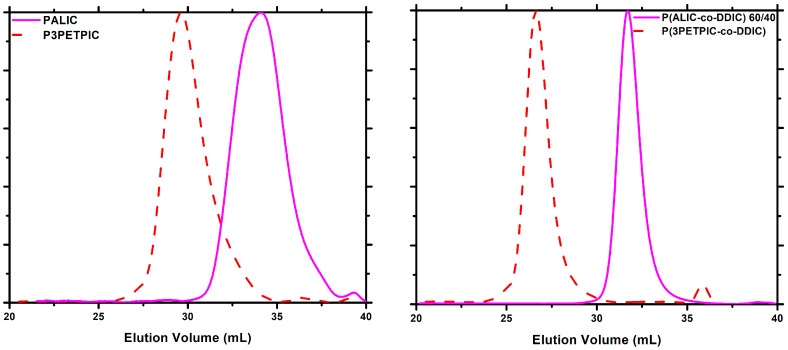
SEC traces of the thiol-ene click reaction products.

A thiol-ene click reaction was performed with 2-phenylethanethiol to obtain the corresponding functional copolymer-bearing side phenyl groups, utilizing the pendant double bond of the monomeric unit of ALIC. The purpose of this modification was to enhance the thermal stability of polyisocyanates, since the phenyl moieties offer thermal stability [77]. The click reaction was tested on both the PALIC homopolymer and the copolymer P(ALIC-co-DDIC) 60/40 and was conducted using AIBN at 50 °C in dichloromethane, as reported in the literature [63] (Scheme 5). The success of the reaction in both cases was confirmed by ^1^HNMR spectroscopy. The appearance of signals within the 7.0–7.5 ppm range was indicative of the presence of aromatic protons in the polymer and confirmed the success of the functionalization reaction. The integration of the aforementioned peak, along with the peaks observed at 5.10–5.36 ppm and 5.70 ppm, which correspond to the vinyl protons of the unreacted ALIC monomeric units, enabled the calculation of the incorporation rate of the novel functional group. The introduction of the phenylethyl group was achieved to a satisfactory degree, since the incorporation rate was calculated at 89% in the copolymer and 61% in the homopolymer, respectively (Table S3 of the SIS). The characteristic NMR spectra are given in Figure 6 and Figure S8. The SEC traces of the functionalized polymers, depicted in Figure 7**,** confirm the increase in the molecular weight of the polymers while maintaining notably low distributions. Furthermore, no obvious degradation or crosslinking side reactions were observed during the thiol-ene post-polymerization reaction. These encouraging results allow for the further chemical transformation of these copolymers, thus facilitating the synthesis of more complex macromolecular architectures with modified properties.

### 3.7. Thermal Analysis

#### 3.7.1. DSC Analysis

**Figure 8:**
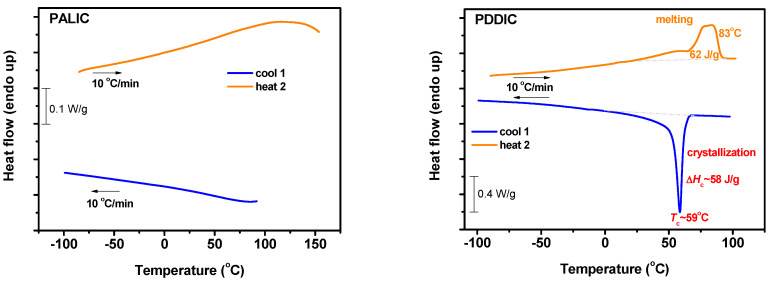
DSC thermograms for PALIC (**left**) and PDDIC (**right**) homopolymers.

The thermal properties of the samples were studied by differential scanning calorimetry, DSC, measurements. Due to their thermal sensitivity, the homopolymers and the copolymers were heated for the annealing procedure up to 100 °C, and the second heating results were obtained. The data for the homopolymers are given in Figure 8. There are no detailed previous results in the literature for the specific homopolymers. For PALIC, no obvious transition was observed in the temperature range from −100 to 100 °C. It is well known that PICs adopt helical structures both in solution and in the solid state. The absence of any signal in the DSC thermogram indicates that local motions are not allowed, so there is no Tg in the homopolymer, and the formation of crystalline structures is prohibited due to the small size of the side allyl groups. These findings reveal that the specific polymer behaves as a “molecular ruler”, or, in other words, that the helical structure does not change direction in space, introducing some kind of flexibility in the molecule. The rather low molecular weight of the sample (M_n_ = 11000 by SEC) facilitates the formation of this “molecular ruler”.

The thermal behavior of PDDIC was different. As was expected, the long side chain of the monomer unit gave rise to side chain crystallization [79,80]. It has been reported in the literature from the homologous series of several polymers with long alkyl side groups, such as poly(α-olefins), polymethacrylates, polyacrylates, polyacrylamides, etc., that when the side group of the monomer contains 12 carbon atoms or more, then side chain crystallization takes place. Therefore, the PDDIC exhibits a cooling crystallization exotherm with Tc = 59 °C and crystallization enthalpy ΔHc = 58 J/g and on a heating melting endotherm with Tm = 83 °C and melting enthalpy ΔHm = 62 J/g.

**Figure 9:**
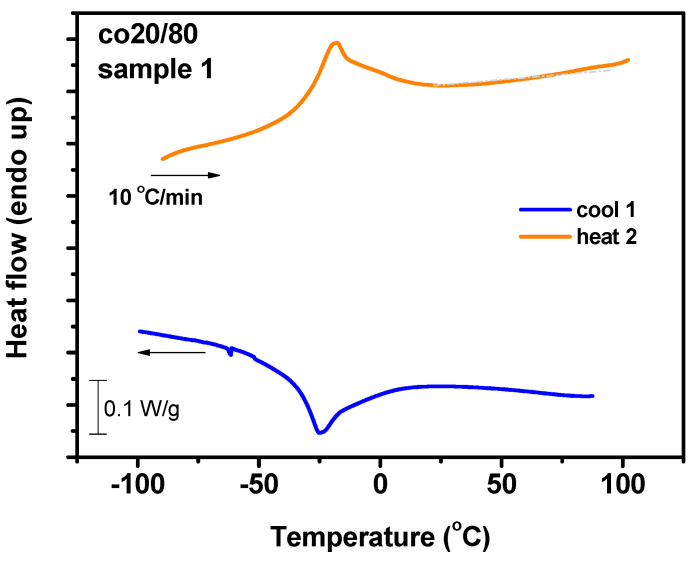
DSC thermograms for the P(ALIC-co-DDIC) statistical copolymer.

The DSC thermograms for the statistical copolymers are displayed in Figure 9 and Figure S9 of the SIS. As in the cases of the respective homopolymers, no glass transition was observed for the statistical copolymers in the temperature range examined. The statistical copolymer with the highest composition in DDIC exhibited a melting endotherm upon heating and a crystallization exotherm upon cooling. However, both the Tm and Tc values were much lower than those obtained for the PDDIC homopolymers. The multiblock structure of the statistical copolymer in combination with the high content in DDIC permitted the organization of crystalline domains in the statistical copolymers. However, the interference of the ALIC monomer sequences restricted the organization of the crystalline phases, leading to much lower Tm and Tc values (−20 and −25 °C, respectively). Upon decreasing the DDIC content in the statistical copolymers, the crystallization was drastically inhibited by the helical domains of the ALIC sequences. This was also promoted by the fact that the mean sequence length of ALIC was higher than that of DDIC due to the higher reactivity ratio of ALIC compared to that of DDIC. Furthermore, these small traces of melting or crystallization moved progressively to even lower temperatures so that, for the 80-20 sample, no transition was obvious in the thermogram.

**Figure 10:**
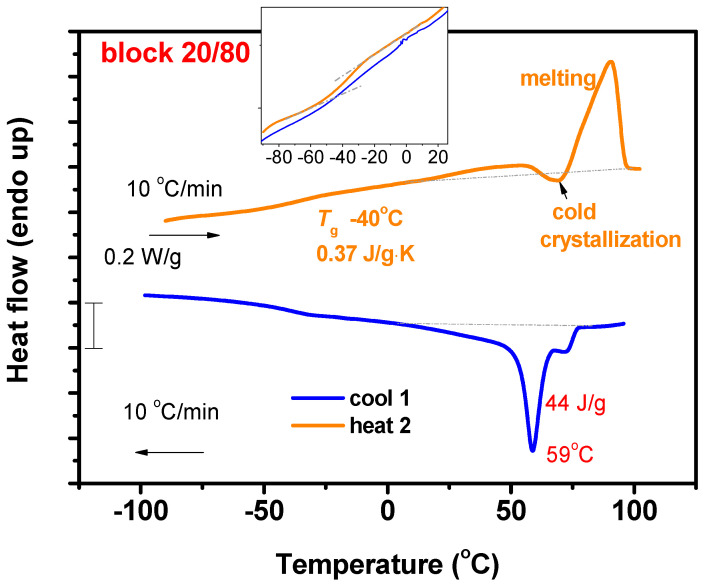
DSC thermograms for the P[DDIC-b-(DDIC-co-ALIC)] block copolymer.

A different situation was obtained for the P[DDIC-b-(DDIC-co-ALIC)] copolymers. The presence of a pure block of the crystalline block of PDDIC gave rise to the formation of crystalline domains, as shown in Figure 10 and Figure S10 of the SIS. The copolymer with the highest content in DDIC showed similar behavior as the PDDIC homopolymer, except that both the enthalpies of melting and crystallization were slightly lower than those of the homopolymer due to the minor restrictions derived from the second block for the organization of the crystalline phases. The decrease in the DDIC content led to a different behavior. The first heating procedure showed, as in the previous sample, the characteristic melting endotherm progressively at lower temperatures with decreasing DDIC content. However, the cooling run, and, therefore, the subsequent second heating run, showed almost an absence of crystallization and melting, respectively. This is direct evidence of the slower kinetics of crystallization of the PDDIC blocks compared to the respective homopolymers, or in other words, it reveals that the second statistical block P(DDIC-co-ALIC) impedes the nucleation process and therefore affects the crystallization behavior. Another interesting finding for the block copolymers was the appearance of a weak glass transition at temperatures between −31 and −40 °C. These transitions are attributed to the second blocks of the structures, which are statistical copolymers of DDIC and ALIC. The molecular weights of these samples were relatively low, and therefore, the sequences of the ALIC monomer units were very short. These short sequences did not have the possibility to adopt a clear helical structure and, consequently, were more flexible, giving rise to these weak transitions at low temperatures. In conclusion, the macromolecular structure affects the thermal behavior of these polymers to a great extent.

#### 3.7.2. Thermal Decomposition

##### Homopolymers

**Figure 11:**
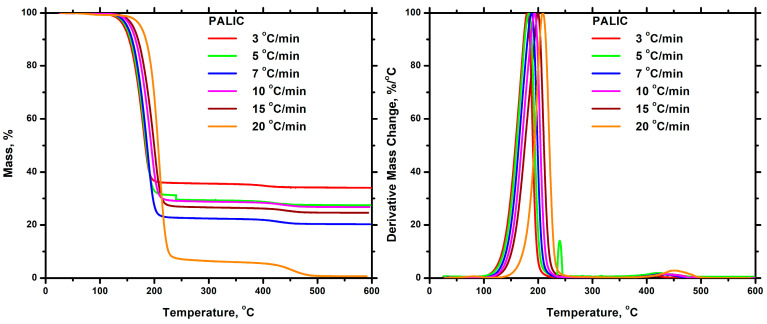
TGA (**left**) and DTG (**right**) plots for the PALIC at all heating rates.

**Figure 12:**
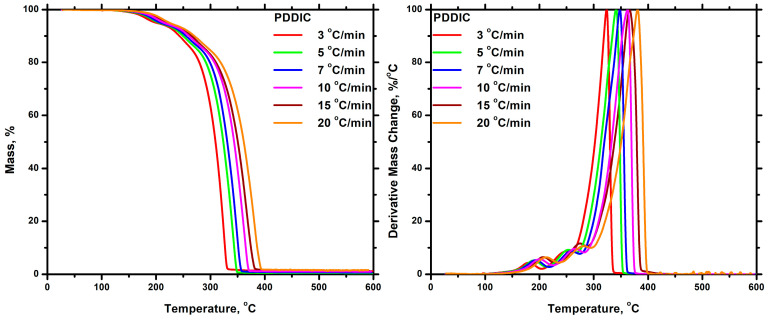
TGA (**left**) and DTG (**right**) plots for the PDDIC at all heating rates.

The thermal degradation of each homopolymer was investigated using TGA and DTG techniques. The measurements were performed at various heating rates (3, 5, 7, 10, 15, and 20 °C/min). The thermograms obtained from TGA and DTG for the homopolymers are presented in Figure 11 and Figure 12. A comparison of the DTG diagrams revealed that PDDIC exhibited greater thermal stability than PALIC. The thermal degradation of PALIC started at a lower temperature than that of PDDIC, and the peak corresponding to the maximum degradation rate occurred at significantly lower temperatures as well. The complete thermal degradation of PDDIC took place in a temperature range of 200 to 250 °C, higher than that of PALIC. Moreover, PALIC exhibited a single, relatively symmetric degradation peak in DTG, whereas PDDIC displayed two additional but minor peaks. This indicates that the degradation mechanism is relatively simple for PALIC, whereas in the case of PDDIC, it is more complex, involving additional degradation steps. However, the highest proportion of PDDIC was degraded during the third stage at elevated temperatures. The increased thermal stability of PDDIC can be attributed to its semi-crystalline nature and, therefore, the strong intermolecular interactions developed between the polymer chains. In each case, it is evident that the temperature values corresponding to the initiation and completion of thermal degradation, as well as the temperature at the maximum rate of degradation, increased upon the application of a higher heating rate. The thermal degradation process is a kinetic phenomenon, which explains the slower response of the system to the temperature change as the heating rate increased. A characteristic finding of the thermal decomposition of PALIC was the appearance of a high amount of residue. It seems that the presence of the side allyl moieties of PALIC led to extended carbonization phenomena.

##### Statistical Copolymers

**Figure 13:**
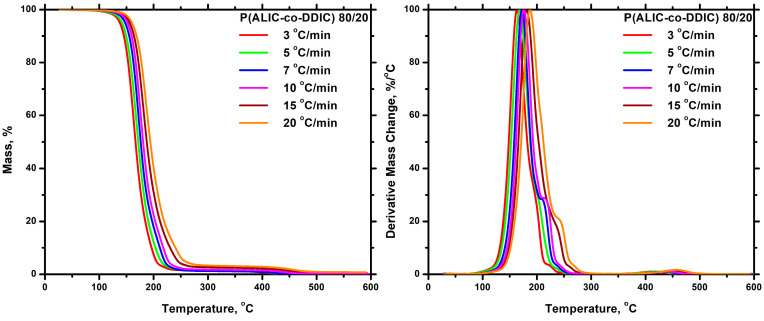
TGA (**left**) and DTG (**right**) plots for the P(ALIC–co–DDIC) 80/20 at all heating rates.

The thermal degradation of each statistical copolymer was also investigated at various heating rates. Characteristic thermograms are displayed in Figure 13, whereas additional data are included in the SIS (Figures S11–S14). In a similar manner to the homopolymers previously discussed, the initiation and completion temperatures of the thermal degradation, along with the temperature at the maximum rate of degradation, increased with increasing heating rates in the copolymers. In fact, an increase in the heating rate resulted in a greater distinction between the stages of thermal degradation.

Τhe degradation mechanism of the statistical copolymers is complex and depends on the composition of each sample. The thermal decomposition profiles of the statistical copolymers exhibited a combination of characteristics observed in the degradation behavior of both the PALIC and PDDIC homopolymers. As suggested above, the reactivity ratios were measured as r_ALIC_ = 2.64 and r_DDIC_ = 1.14. This means that PALIC sequences were more favored than PDDIC, resulting in the thermal degradation behavior of the copolymers being more influenced by PALIC.

In the P(ALIC-co-DDIC) 20–80 sample, the primary thermal degradation peak observed in the DTG plot was in close proximity to that of PDDIC, which can be attributed to the higher composition in DDIC monomeric units in this polymer. In the case of P(ALIC-co-DDIC) 40–60, where the content of PALIC was increased, the two peaks of the main thermal decomposition stages were obvious. At low heating rates, a third peak emerged between the two basic stages, which at higher heating rates took the form of a shoulder. This peak was due to the multiblock structure, which, as mentioned earlier, resulted from the reactivity ratios of the monomers. Finally, in the P(ALIC-co-DDIC) 80–20 sample, the main degradation event took place at lower temperatures, similarly to PALIC homopolymer. This led to the conclusion that an increase in the proportion of PALIC in the copolymer would result in a corresponding increase in the system’s thermodynamic instability. A final point to be mentioned is the absence of any appreciable amount of residue during the thermal decomposition of the statistical copolymers, meaning that carbonization was prohibited in the presence of the sequences of DDIC.

#### 3.7.3. Kinetics of Thermal Degradation of Homopolymers and Statistical Copolymers

Ozawa–Flynn–Wall (OFW) [81,82,83] and Kissinger–Akahira–Sunose (KAS) [84] methodologies were applied for the measurement of the activation energies, E_a_, of the thermal decomposition of the homopolymers and the statistical copolymers, as explained in detail in the SIS. These isoconversional methods, which are also considered model-free, do not necessitate any prior knowledge of the degradation mechanism in order to determine the activation energy values [85]. Both approaches are particularly beneficial for analyzing thermogravimetric data from complex chemical processes, and their implementation does not require an understanding of the reaction order governing the decomposition or the underlying degradation mechanism. Also, it is worthy to mention that the OFW method is based on the Doyle approximation [86], whereas the KAS method is founded upon the Coats–Redfern approximation [87], which offers greater precision. Consequently, the latter approach is regarded as a more accurate approach for the determination of the activation energy associated with the thermal decomposition process.

In order to obtain kinetic parameters from thermogravimetric data, both the OFW and KAS methods require the measurement of temperature values corresponding to specific fixed values of conversion, α, which are determined from experiments conducted at varying heating rates, β. The plots of the OFW and KAS methods are shown in the SIS (Figures S15–S21), and the activation energy values obtained for the homopolymers as well as the copolymers are given in Table 5, respectively.

A comparison of the results obtained by the two methods reveals that the E_a_ values obtained by the KAS method were slightly lower than those obtained by OFW for both homopolymers. In the PALIC homopolymer, the activation energy values (E_a_) demonstrated an increase with an increasing degradation rate. In contrast, the PDDIC values exhibited a decrease. This could be explained by the fact that, in the case of PALIC, early stages of degradation require less energy and, most probably, involve the depolymerization of the macromolecular chain from the chain-end through the formation of cyclotrimers. However, as the process continues, higher E_a_ values are needed, since carbonization takes place with the implication of the remaining double bonds of the allyl moieties. Contrary to this, in PDDIC, degradation becomes more facile as the process progresses. Furthermore, the E_a_ values for PALIC are found to be lower for lower conversion rates (a) compared to PDDIC, which is consistent with the observation that PALIC begins to degrade at lower temperatures.

As far as statistical copolymers are concerned, the highest dependence of the E_a_ values on the degradation rate was found in the P(ALIC-co-DDIC) 60/40 sample. In the P(ALIC-co-DDIC) 40/60 sample, the values of E_a_ decreased until 40% of degradation had occurred. Concurrently, in the P(ALIC-co-DDIC) 50/50 sample, the activation energy also exhibited reducing values until 50% of the degradation process was completed. This observation is in accordance with the molar ratio of the monomeric units in each sample, respectively. In contrast to the behavior observed in PALIC, the E_a_ values in the P(ALIC-co-DDIC) 80/20 sample decreased as thermal degradation proceeded. This suggests that the random placement of DDIC sequences along the main macromolecular chain exerted a significant influence on the thermal degradation mechanism. Regarding the average values of the activation energies of the statistical copolymers, it can be concluded that more or less similar E_a_ values were obtained for all copolymers without substantial differentiation. The highest values were obtained for the 20/80 sample, with the highest content of the most thermally stable DDIC. It has to be mentioned that, for all copolymers, the E_a_ values were slightly lower than those measured for the corresponding homopolymers, reflecting the absence of any carbonization effect, which caused the incomplete degradation of PALIC and the increased E_a_ values for this homopolymer at the latter stages of decomposition.

To further investigate this topic, an attempt was made to predict the PALIC and PDDIC decomposition mechanism by comparing the E_a_ values calculated by the OFW and KAS methods with those derived from 41 different mathematical models, which are reported in the literature. Each mathematical model, which is expressed as an algebraic function, represents a potential mechanism underlying the decomposition reaction. In this context, algebraic expressions of functions representing the most commonly occurring reaction mechanisms in solid-state reactions are presented in the SIS (Tables S4 and S5) [88]. The plots demonstrating the greatest linearity and closest agreement in E_a_ values calculated from the OFW and KAS methods were selected as the proposed mechanisms for the decomposition of the two homopolymers.

**Figure 14:**
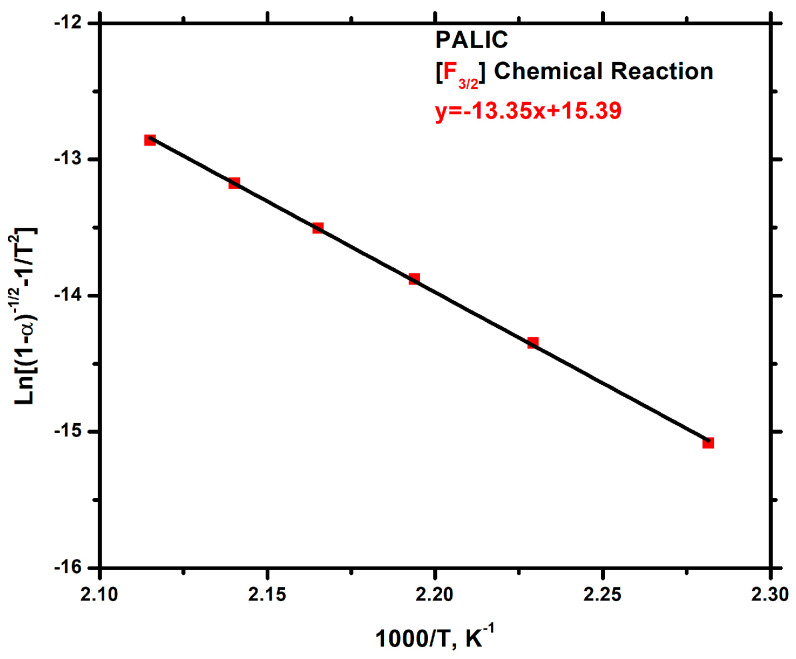
Chemical reaction degradation mechanism plot for the PALIC.

Among the various models for the decomposition of the PALIC homopolymer, equation F_3/2_ demonstrates excellent linearity, and the calculated E_a_ value is in close proximity to the E_a_ value derived from the KAS method, which is more accurate than the OFW method. The suggested mechanism belongs to the category of chemical reactions, and, according to the mathematical model of the thermal degradation mechanism F_3/2_, the activation energy is equal to 110.86 KJ/g. The plot is given in Figure 14.

**Figure 15:**
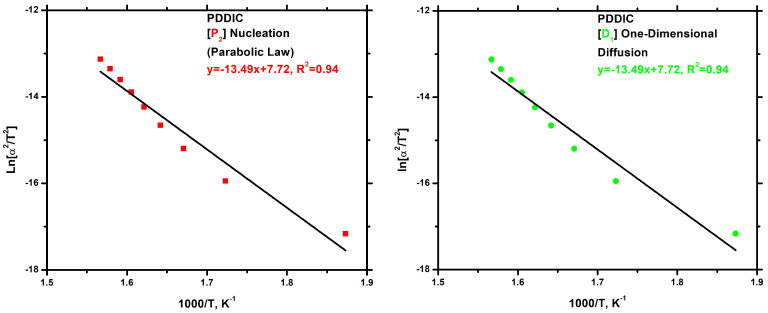
Nucleation (Parabolic Law) (**left**) and one-dimensional diffusion (**right**) degradation mechanism plots for the PDDIC.

As far as the PDDIC homopolymer is concerned, the P_2_ and D_1_ mathematical models were proposed as the underlying mechanisms, similarly to the approach adopted previously. The P_2_ model represents a nucleation process occurring during the thermal degradation of the material, while the D_1_ model is indicative of one-dimensional diffusion. It is notable that, in both cases, the activation energy values were identical, equal to 112.10 KJ/g. The plots are displayed in Figure 15.

In conclusion, the different decomposition patterns of the two homopolymers are reflected by the different degradation mechanisms, as was revealed by the previous discussion.

#### 3.7.4. Block Copolymers

**Figure 16:**
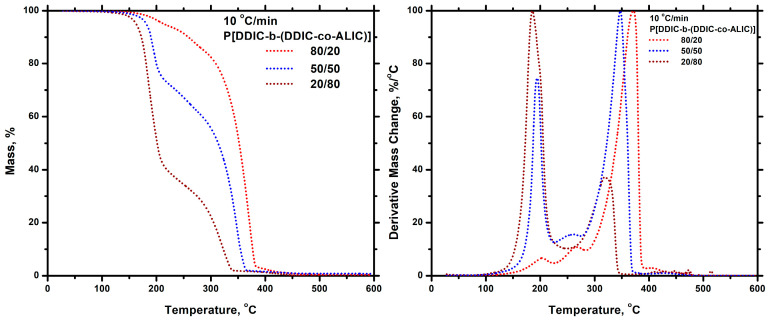
TGA (**left**) and DTG (**right**) plots for all the P[(DDIC–b–(DDIC-co-ALIC)] at 10 °C/min.

**Figure 17:**
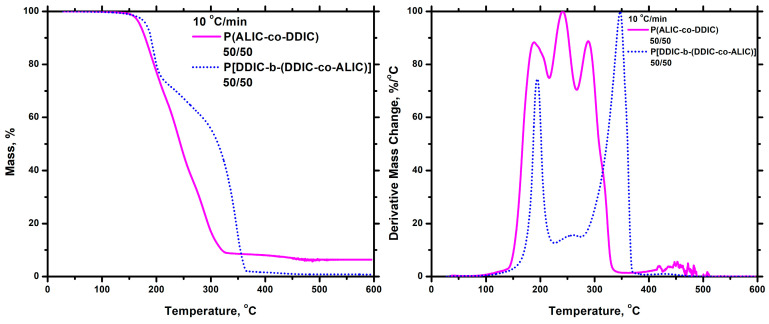
TGA and DTG plots for the ALIC/DDIC 50/50 Statistical and Block Copolymer at 10 °C/min.

The thermal degradation of the block copolymers was also examined utilizing the techniques of thermogravimetric analysis (TGA) and differential thermogravimetry (DTG). The measurements were conducted at a heating rate of 10 °C/min, and the resulting differential thermogravimetric (DTG) diagrams are displayed in Figure 16. For all three samples, the same degradation pattern was observed. The decomposition took place with two distinct steps. The one at a lower temperature corresponded to the degradation of the ALIC sequences, and the other at a higher temperature to the pure PDDIC block. This is obvious if the DTG traces of the block copolymers are compared with the respective homopolymers. This is undoubtedly proven by comparing the thermograms of block copolymer 50/50 with those of the homopolymers PALIC and PDDIC at the same heating rate of 10 °C/min, as shown in the SIS (Figure S22). The mass loss at each degradation step was in perfect agreement with the composition of the copolymers. This behavior is the result of the different thermal stability of the various components of the blocks (PALIC and PDDIC sequences) and the structure of the copolymers. The thermal decomposition pattern of these block copolymers is completely different compared to the respective statistical copolymers. This is shown in Figure 17, where the DTG traces of the statistical and the block copolymer with the same composition (50/50) are given. Consequently, the macromolecular architecture plays a crucial role, dramatically affecting the thermal degradation behavior of the copolymers.

#### 3.7.5. Thiol-Ene Products

**Figure 18:**
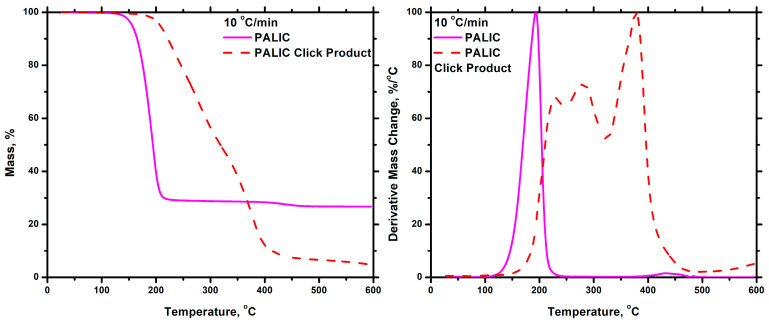
TGA (**left**) and DTG (**right**) plots for the PALIC and the click Product at 10 °C/min.

**Figure 19:**
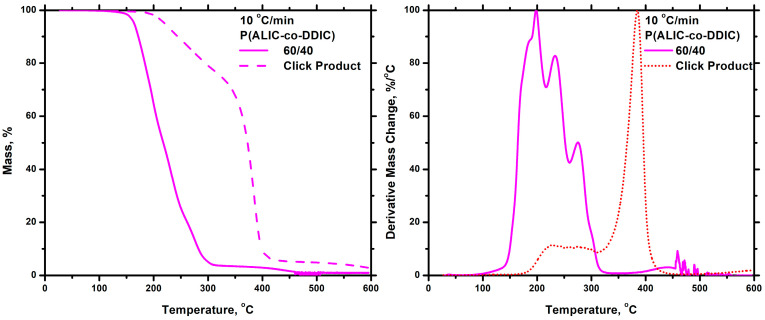
TGA and DTG plots for the P(ALIC–co–DDIC) 60/40 and click product at 10 °C/min.

The effect of the click reaction, with the incorporation of the aromatic groups along the polyisocyanates chain, on the thermal stability of the PALIC homopolymer and the P(ALIC-co-DDIC) 60/40 statistical copolymer was studied by TGA measurements carried out at a heating rate of 10 °C/min. The thermograms prior to and after the click reaction are shown in Figure 18 and Figure 19 for the homopolymer and the statistical copolymer, respectively. As previously stated, the PALIC homopolymer was thermally unstable, exhibiting degradation at relatively low temperatures. However, the product of the click reaction in PALIC displayed enhanced thermal stability, with the main degradation peak appearing near 400 °C. Two additional minor peaks were observed at slightly lower temperatures, attributed to the presence of unreacted ALIC monomeric units, since the yield of the click reaction was 60%. A similar observation was made in the case of the click reaction product in sample P(ALIC-co-DDIC) 60-40. The thermal stability was even more pronounced in this case due to the higher yield of the click reaction, which was close to 90%. The substantial enhancement of the thermal stability of the products can be attributed to the successful incorporation of the phenyl groups on the polymer structure. Therefore, these post-polymerization reactions may offer the possibility to manipulate the thermal stability of polyisocyanates.

These copolymers can be further employed as precursors for the synthesis of various functionalized materials based on PICs. The introduction of the functional groups can be achieved through the performance of addition reactions to the pendant double bonds of the allyl moieties. With these reactions, it would be possible to manipulate the solution and solid-state properties of the parent materials, as well as to afford materials with novel properties. In addition, using thiol-end-functionalized polymers and click chemistry, it would be feasible to synthesize new, well-defined graft and block–graft copolymers.

## 4. Conclusions

Coordination polymerization techniques were employed for the synthesis of statistical copolymers of n-dodecyl isocyanate, DDIC, and allyl isocyanate, ALIC, using the chiral half-titanocene complex CpTiCl_2_(O-(S)-2-Bu) as an initiator. Samples with narrow molecular weight distribution and relatively high molecular weights were obtained. The monomer reactivity ratios of the statistical copolymers were calculated assuming that the terminal model applies for the copolymerization system. Both linear graphical methods and the computer program COPOINT were used, revealing the existence of a mutiblock structure with longer ALIC than DDIC sequences. The distribution of the monomer dyads along with their mean sequence lengths were also calculated, confirming the previously reported results. The thermal properties of these samples were studied by differential scanning calorimetry, DSC, measurements, showing that PDDIC is a semicrystalline polymer, whereas PALIC adopts a stable helical structure without showing any clear thermal transition between −100 and 100 °C. It was also found that the multiblock structure of the statistical copolymers and the relatively small mean sequence length of the DDIC sequences restricted the crystallization. The kinetics of the thermal decomposition of the statistical copolymers were studied by thermogravimetric analysis, TGA, and differential thermogravimetry, DTG, and the activation energy of this process was calculated by employing several theoretical models. It was found that the respective homopolymers followed a different degradation mechanism and showed a large difference in thermal stability, with PDDIC being much more thermally stable than PALIC. Block copolymers P[DDIC-b-(DDIC-co-ALIC)] were also synthesized by sequential addition of monomers and coordination polymerization methodologies. The samples were characterized by nuclear magnetic resonance, NMR, spectroscopy; size exclusion chromatography, SEC; and DSC. The thermal stability of the blocks was also studied by TGA and DTG and compared to the corresponding statistical copolymers. It was clear that the crystallization was less restricted for these samples due to the presence of the pure PDDIC block, whereas the thermal degradation took place mainly in two steps corresponding to the degradation profiles of the respective homopolymers. The thiol-ene click post-polymerization reaction was performed to introduce aromatic groups along the polyisocyanate chain. The reaction was highly efficient and afforded products with considerably improved thermal stability compared to their parent materials.

## Figures and Tables

**Table 1:** PALIC samples from kinetic analysis.

Time (min)	M_n_^a^	M_w_^a^	Đ^a^	Yield (%)
15	3169	3607	1.14	10.0
30	4328	5836	1.35	39.2
45	4830	7012	1.45	41.6
60	5336	8096	1.52	42.6

^a^ by SEC in CHCl_3_.

**Table 2:** Molecular characteristics of the statistical copolymers P(ALIC-co-DDIC).

Feed Molar Ratio ALIC/DDIC	Yield (%)	Molar Ratio ALIC/DDIC ^a^	M_w_^b^	Đ ^b^
80/20	34.0	90/10	19800	1.06
60/40	35.3	73/27	17600	1.07
50/50	30.5	63/37	16800	1.06
40/60	38.5	51/49	21600	1.09
20/80	42.7	23/77	26100	1.08

^a^ by ^1^H NMR in CDCl_3_. ^b^ by SEC in CHCl_3_.

**Table 3:** Reactivity ratios of the statistical copolymers P(ALIC-co-DDIC).

Method	r_ALIC_	r_DDIC_
F-R	2.63	1.14
IF-R	2.60	1.13
K-T	2.60	1.12
ext K-T	3.04	1.08
COPOINT	2.64	1.14

**Table 4:** Molecular characteristics of the block copolymers P[DDIC-b-(ALIC-co-DDIC)].

Feed Molar Ratio ALIC/DDIC	Molar Ratio ALIC/DDIC ^a^	Yield (%)	M_w_^b^	Đ ^b^
80/20	76/24	42.8	9200	1.11
50/50	44/56	38.0	11100	1.17
20/80	8/91	41.7	8300	1.13

^a^ By ^1^H NMR in CDCl_3_; ^b^ By SEC in CHCl_3._

**Table 5:** Activation energies, Ea, for the homopolymers PALIC and PDDIC and the statistical copolymers P(ALIC-co-DDIC).

Weight Loss (a)	PALIC		PDDIC		20/80		40/60		50/50		60/40		80/20	
	OFW	KAS	OFW	KAS	OFW	KAS	OFW	KAS	OFW	KAS	OFW	KAS	OFW	KAS
0.1	94.56	87.26	162.04	153.24	136.95	129.14	117.67	110.11	111.94	104.46	143.26	135.95	122.32	115.18
0.2	102.13	94.65	135.12	125.65	113.51	105.12	111.02	103.13	107.78	100.05	128.22	120.66	122.49	115.26
0.3	110.94	103.29	121.49	111.77	109.03	100.30	96.15	87.84	100.63	95.65	124.15	116.42	122.66	115.26
0.4	120.49	112.77	116.42	106.45	112.85	103.71	93.16	84.60	88.34	80.12	119.33	111.44	121.24	113.85
0.5	130.55	122.82	113.76	103.71	111.69	102.30	96.89	88.00	84.76	76.26	104.71	96.64	118.33	110.77
0.6	145.43	137.53	112.27	102.05	110.77	101.22	99.97	90.83	88.50	79.77	96.31	88.09	113.60	106.04
0.7			110.86	100.55	111.44	101.71	99.72	90.41	92.99	83.931	95.07	86.59	107.03	99.30
0.8			109.28	98.89	112.68	102.71	100.14	90.58	92.90	83.60	99.39	90.66	100.14	92.24
0.9			106.20	95.81	111.69	101.63	101.88	92.07	95.81	86.09	105.54	96.40	84.60	76.44

## Data Availability

The original contributions presented in the study are included in the article; further inquiries can be directed to the corresponding author.

## Supplementary Materials

The following supporting information can be downloaded at: https://www.mdpi.com/article/10.3390/polym16243537/s1, The SIS contains basic theory for the calculation of the reactivity ratios of the monomers and the kinetics of thermal decomposition. The following figures are also included: Figure S1: SEC traces of PALIC homopolymers from the kinetic experiments. Figure S2: SEC traces of PDDIC homopolymer. Figure S3: F–R plot. Figure S4: inv. F–R plot. Figure S5: K–T plot. Figure S6: ext. K–T plot. Figure S7: ^1^H NMR spectrum of the sample P[DDIC–b–(DDIC-co-ALIC)] 20/80 in CDCl_3_. Figure S8: ^1^H NMR spectrum of the sample PALIC Click product in CDCl_3_^.^ Figure S9: DSC traces for the statistical copolymers P(ALIC-co-DDIC) 50/50, 60/40 and 80/20. Figure S10: DSC traces of the block copolymers P[DDIC-b-(DDIC-co-ALIC)] 20/80 and 80/20 block copolymers. Figure S11: TGA (left) and DTG (right) plots for the P(ALIC–co–DDIC) 20/80 at all heating rates. Figure S12: TGA (left) and DTG (right) plots for the P(ALIC–co–DDIC) 40/60 at all heating rates. Figure S13: TGA (left) and DTG (right) plots for the P(ALIC–co–DDIC) 50/50 at all heating rates. Figure S14: TGA (left) and DTG (right) plots for the P(ALIC–co–DDIC) 60/40 at all heating rates. Figure S15: OFW and KAS plots for the P(ALIC-co-DDIC) 20/80 statistical copolymer. Figure S16: OFW and KAS plots for the P(ALIC–co–DDIC) 50-50 at all heating rates. Figure S17: OFW and KAS plots for the P(ALIC–co–DDIC) 40-60 at all heating rates. Figure S18: OFW and KAS plots for the P(ALIC–co–DDIC) 60-40 at all heating rates. Figure S19: OFW and KAS plots for the P(ALIC–co–DDIC) 80-20 at all heating rates. Figure S20: OFW (left) and KAS (right) plots for the PALIC. Figure S21: OFW (left) and KAS (right) plots for the PDDIC. Figure S22: DTG plots for the PALIC and DDIC homopolymers and P[DDIC-b-(DDIC-co-ALIC)] 50/50 block copolymer at 10 °C. The following tables are included in the supporting information section: Table S1: Parameters for the synthesis of the statistical copolymers P(ALIC-co-PDDIC). Table S2: Dyad sequences and mean sequence lengths for the statistical copolymers P(ALIC-co-PDDIC). Table S3: Molecular weights of the products of the click reactions. Table S4: Models for the thermal decomposition of PALIC. Table S5: Models for the thermal decomposition of PDDIC. References [89,90,91,92,93,94] are cited in the supplementary materials.
