# Supplementary material for: Statistical and Block Copolymers of n-Dodecyl and Allyl Isocyanate via Titanium-Mediated Coordination Polymerization: A Route to Polyisocyanates with Improved Thermal Stability
Source: Polymers (Basel). 2024 Dec 19;16(24):3537. doi: 10.3390/polym16243537 (PMC11678465; doi:10.3390/polym16243537)
Supplement: Supplementary file 1 [file polymers-16-03537-s001.zip › polymers-3371708-supplementary.pdf]

polymers

Supporting Information

Statistical and Block Copolymers of n-Dodecyl and Allyl Isocyanate via Titanium-Mediated Coordination Polymerization: A Route to Polyisocyanates with Improved Thermal Stability

Maria Iatrou [1], Aikaterini Katara [1], Panagiotis A. Klonos [2], Apostolos Kyritsis [2] and Marinos Pitsikalis [1,*]

[1] Industrial Chemistry Laboratory, Department of Chemistry, National and Kapodistrian University of Athens, Panepistimiopolis Zografou, 15771 Athens, Greece; iatmaria97@gmail.com (M.I.); katerinakatara01@gmail.com (A.K.)
[2] Dielectrics Group, Physics Department, School of Applied Mathematical and Physics Science, National Technical University of Athens, 9 Heroon Polytechniou, GR-15780 Zografou, Greece; panos48al@gmail.com (P.A.K.); akyrits@central.ntua.gr (A.K.)
* Correspondence: pitsikalis@chem.uoa.gr; Tel.: +30-210-727-4768

Reactivity Ratios and Statistical Analysis

The monomer reactivity ratios were estimated using the Fineman–Ross[S1], FR; inverted Fineman–Ross[S1], inv-FR; Kelen–Tüdos (KT)[S2]; and extended Kelen–Tüdos[S2], ext-KT, graphical methods.

According to the Fineman–Ross approach, the reactivity ratios r_{ALIC} and r_{DDIC} of the monomers were calculated by the equation:

$$G = Hr_{ALIC} - r_{DDIC} \quad \text{(S1)}$$

where and G and H are defined as:

$$G = \frac{X(Y-1)}{Y} \quad \text{(S2)}$$

and

$$H = \frac{X^2}{Y} \quad \text{(S3)}$$

M_{ALIC} and M_{DDIC} are the monomer molar feed ratios and $d[M_{ALIC}]$ and $d[M_{DDIC}]$ are the copolymer's composition, calculated by the ^{1}H NMR spectra. The parameters X and Y are given as:

$$X = \frac{M_{ALIC}}{M_{DDIC}} \quad \text{(S4)}$$

and

$$Y = \frac{d[M_{ALIC}]}{d[M_{DDIC}]} \quad \text{(S5)}$$

According to this equation, G varies linearly with H. The slope of the line is equal to the ratio r_{ALIC}, while the intercept is equal to the ratio r_{DDIC}.

The inv. F–R method is expressed by the equation:

 https://doi.org/10.3390/xxxxx

$$\frac{G}{H} = r_{ALIC} - \frac{1}{H} r_{DDIC} \quad \text{(S6)}$$

In this case, the plot of the G/H versus 1/H generates the reactivity ratios r_{HIC} and r_{PEIC} from the intercept and the slope, respectively.

The K–T method can be summarized by the following equation:

$$\eta = \left(r_{ALIC} + \frac{r_{DDIC}}{\alpha}\right)\xi - \frac{r_{DDIC}}{\alpha} \quad \text{(S7)}$$

The η and ξ variables are functions of the G and H parameters and are defined as follows:

$$\eta = \frac{G}{\alpha + H} \quad \text{(S8)}$$

and

$$\xi = \frac{H}{H + \alpha} \quad \text{(S9)}$$

The α variable is a constant, which is equal to $\sqrt{H_{max}H_{min}}$, where H_{max} and H_{min} are the maximum and the minimum H values from the series of measurements, respectively. The plot of the variation of n with ξ is a straight line, and the parameters of $-r_{PEIC}/\alpha$ and r_{HIC} are determined for ξ = 0 and ξ = 1, respectively.

The F-R, inv. F-R, and K-T methods for determining the reactivity ratios are valid for low conversions because they rely on the differential form of the copolymerization equation. When polymerizations are carried out up to high conversions, the composition of the products changes significantly, and thus, the integrated form of the copolymerization equation should be used. Therefore, the ext. K–T is a much better method for determining the reactivity ratios. It is based on the same equations as the conventional method (eq (S7–S9)), modifying the G and H to:

$$G = \frac{Y - 1}{a} \quad \text{(S10)}$$

and

$$H = \frac{Y}{z^2} \quad \text{(S11)}$$

where

$$z = \frac{\log(1 - \zeta_{ALIC})}{\log(1 - \zeta_{DDIC})} \quad \text{(S12)}$$

$$\zeta_{DDIC} = w\left(\frac{\mu + X}{\mu + Y}\right) \quad (13) \quad \text{(S13)}$$

and

$$\zeta_{ALIC} = \left(\frac{Y}{X}\right)\zeta_{DDIC} \quad \text{(S14)}$$

μ is the ratio of the molecular weight of DDIC to the molecular weight of ALIC, and w is the conversion of the copolymerization reactions.

In order to determine the statistical distribution of the dyad monomer sequences M_{ALIC}-M_{ALIC}, M_{DDIC}-M_{DDIC}, and M_{ALIC}-M_{DDIC}, the following equations were used[S3]:

$$[M_{\mathrm{ALIC}}\text{-} M_{\mathrm{ALIC}}] = \varphi_{ALIC} - \frac{2\varphi_{ALIC}(1 - \varphi_{ALIC})}{1 + [(2\varphi_{ALIC} - 1)^2 + 4r_{ALIC}r_{DDIC}\varphi_{ALIC}(1 - \varphi_{ALIC})]^{\frac{1}{2}}} \quad \text{(S15)}$$

$$[M_{\mathrm{DDIC}}\text{-}M_{\mathrm{DDIC}}] = (1-\varphi_{ALIC}) - \frac{2\varphi_{ALIC}(1-\varphi_{ALIC})}{1+[(2\varphi_{ALIC}-1)^2+4r_{ALIC}r_{DDIC}\varphi_{ALIC}(1-\varphi_{ALIC})]^{1/2}} \quad \text{(S16)}$$

$$[M_{\mathrm{ALIC}}\text{-}M_{\mathrm{DDIC}}] = \frac{4\varphi_{ALIC}(1-\varphi_{ALIC})}{1+[(2\varphi_{ALIC}-1)^2+4r_{ALIC}r_{DDIC}\varphi_{ALIC}(1-\varphi_{ALIC})]^{1/2}} \quad \text{(S17)}$$

where [M_{ALIC}-M_{ALIC}], [M_{ALIC}-M_{DDIC}], and [M_{DDIC}-M_{DDIC}] are the mole fractions of the corresponding dyads in the copolymer and φ_{ALIC} is the ALIC mole fraction in the copolymer chain. The reactivity ratio values, r_{ALIC} and r_{DDIC}, obtained from COPOINT were employed in the equations. The mean sequence lengths, μ_{ALIC} and μ_{DDIC}, were also calculated using the following equations [S4]:

$$\mu_{ALIC} = 1 + r_{ALIC}\frac{[ALIC]}{[DDIC]} \quad \text{(S18)}$$

and

$$\mu_{DDIC} = 1 + r_{DDIC}\frac{[DDIC]}{[ALIC]} \quad \text{(S19)}$$

Kinetics of Thermal Decomposition

The activation energy, Ea, of the thermal decomposition process was calculated using the isoconversional methods of Ozawa–Flynn–Wall (OFW) and Kissinger–Akahira–Sunose (KAS).

In detail, it is assumed that the reaction rate of the thermal decomposition reaction is expressed as a function of conversion α and temperature T as:

$$\frac{d\alpha}{dt} = f(\alpha)k(T) \quad \text{(S20)}$$

where t is time, α is the conversion of the decomposition reaction, and f(α) is the differential conversion function. The dependance on the temperature can be an Arrhenius equation, that is:

$$k(T) = Ae^{-\frac{E_\alpha}{RT}} \quad \text{(S21)}$$

where A is the pre-exponential factor (min^{-1}), $E\alpha$ is the activation energy, and R is the gas constant (8.314 $J.mol^{-1}.K^{-1}$). Substituting (S21) to (S20) affords:

$$\frac{d\alpha}{dt} = Ae^{-\frac{E_\alpha}{RT}}f(\alpha) \quad \text{(S22)}$$

In case the heating rate β is constant, that is:

$$\beta = \frac{dT}{dt} \quad \text{(S23)}$$

and Equation (S22) is transformed to:

$$\frac{d\alpha}{dT} = \left(\frac{A}{\beta}\right)e^{-\frac{E_\alpha}{RT}}f(\alpha) \quad \text{(S24)}$$

or else:

$$\frac{d\alpha}{f(\alpha)} = \left(\frac{A}{\beta}\right)e^{-\frac{E_\alpha}{RT}}dT \quad \text{(S25)}$$

Upon integrating Equation (S25), the result is the following:

$$g(\alpha) = \int_0^\alpha \frac{d\alpha}{f(\alpha)} = \frac{A}{\beta}\int_{T_0}^{T} e^{\frac{-E_\alpha}{RT}}dT = \frac{AE_\alpha}{\beta R}P(\chi) \quad \text{(S26)}$$

where To and T are the initial and final temperatures of the reaction, respectively. $g(\alpha)$ is the integral conversion function and $x=E\alpha/RT$ [S5-S10]. As is obvious, $g(\alpha)$ depends on the conversion mechanism and its mathematical model. Several algebraic expressions of functions of the most common reaction mechanisms operating in solid-state reactions are given in the literature [S11]. The P(x) function has no analytical solution. Therefore, several approximate expressions have been suggested. Among them are the following:

$$P(\chi) = 0.0048e^{(1.0516\chi)} \quad \text{(S27)}$$

and

$$P(\chi) = \frac{e^{-\chi}}{\chi^2} \quad \text{(S28)}$$

Substitution of Equations (S27) and (S28) to Equation (S26) results in the very well-known Ozawa–Flynn–Wall (OFW) [S12-S14] and Kissinger–Akahira–Sunose (KAS) [S15] equations:

$$\text{OFW:} \quad \ln\beta = \ln\left[\frac{0.0048AE_\alpha}{g(\alpha)R}\right] - 1.0516\frac{E_\alpha}{RT} \quad \text{(S29)}$$

$$\text{KAS:} \quad \ln\frac{\beta}{T^2} = \ln\left[\frac{AR}{g(\alpha)E_\alpha}\right] - \frac{E_\alpha}{RT} \quad \text{(S30)}$$

These methodologies belong to the isoconversional approaches and are "model free" methods, taking into account that the conversion function $f(\alpha)$ is not affected by the change in the heating rate, β, for any values of α. Therefore, plotting lnβ versus 1/T or $\ln(\beta/T^2)$ versus 1/T, respectively, should provide straight lines with slopes directly proportional to the activation energy. Furthermore, if the determined activation energy values do not appreciably vary with various values of α, then a single-step degradation reaction can be concluded.

Rearranging Equation (S30), the following equation is obtained:

$$\ln\frac{g(a)}{T^2} = \ln\left[\frac{AR}{\beta \cdot E_a}\right] - \frac{E_a}{RT} \quad \text{(S31)}$$

The $\ln[g(a)/T^2]$ vs 1000/T graphs are created for a certain value of β (for example, β = 10 °C/min) and for the various proposed models. The slopes of these plots are used to determine Ea values, while the intercepts are used to calculate pre-exponential factors, A. The plot with the best linear fitting and the best agreement of the Ea values determined by the OFW and KAS methods represents the mathematical model or mechanism by which thermal decomposition occurs.

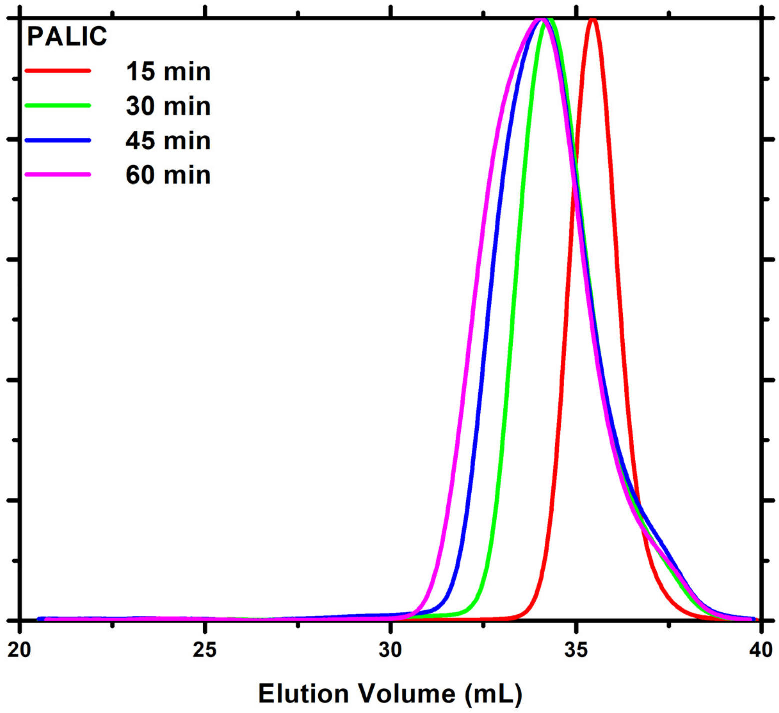

Figure S1. SEC traces of PALIC homopolymers from the kinetic experiments.

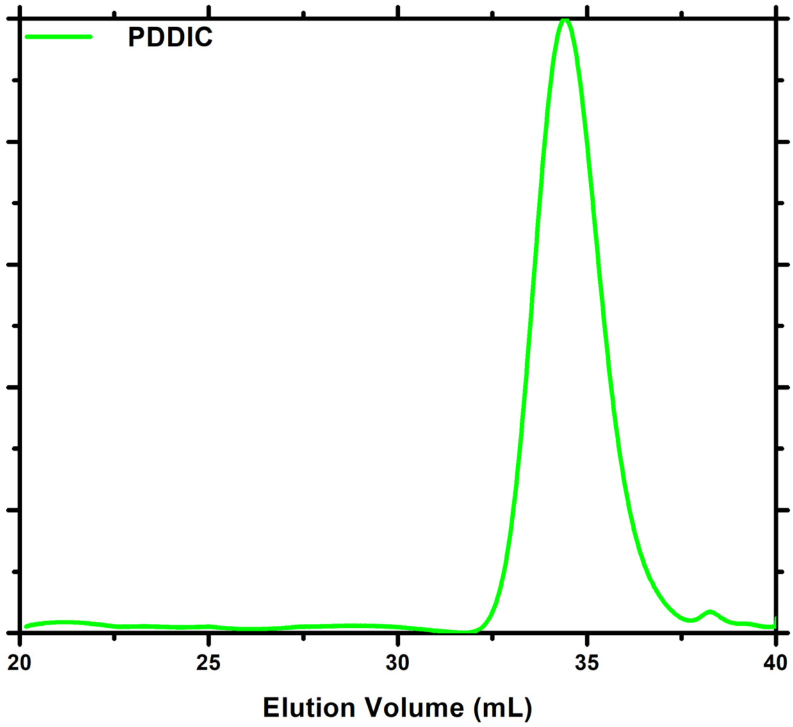

Figure S2. SEC traces of PDDIC homopolymer.

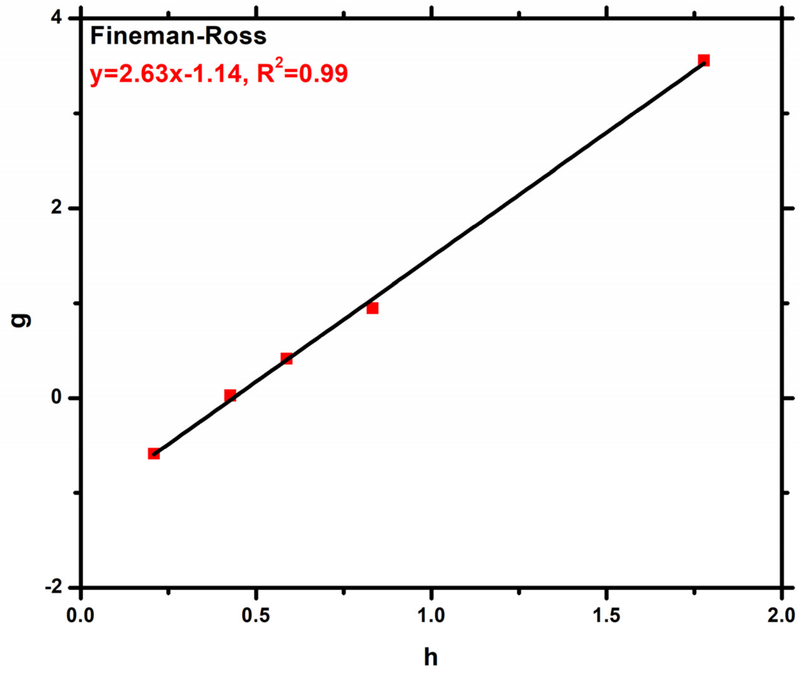

Figure S3. F–R plot.

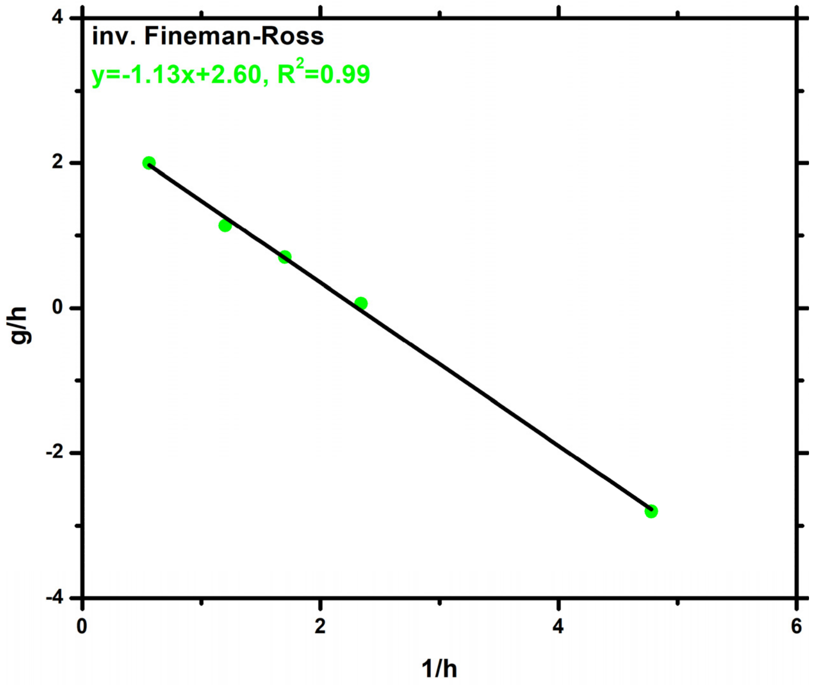

Figure S4. inv. F–R plot.

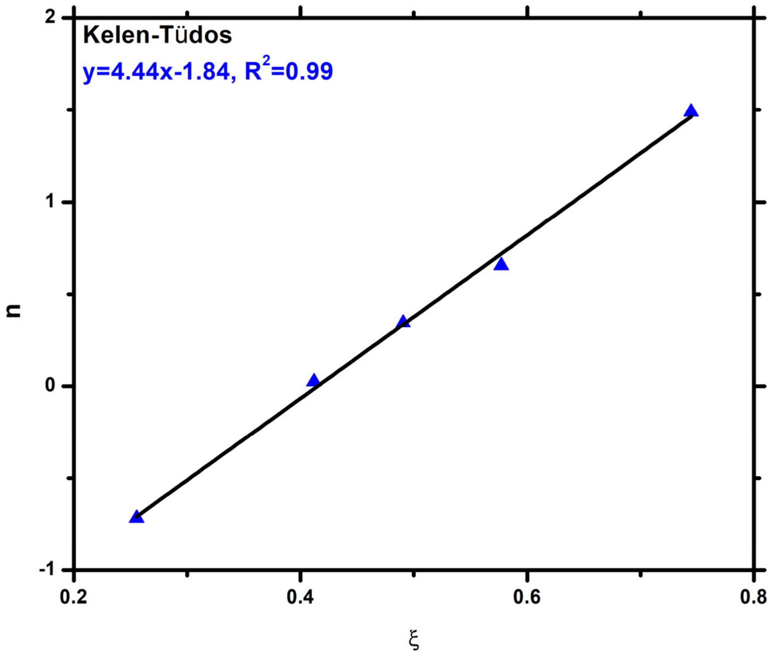

Figure S5. K–T plot.

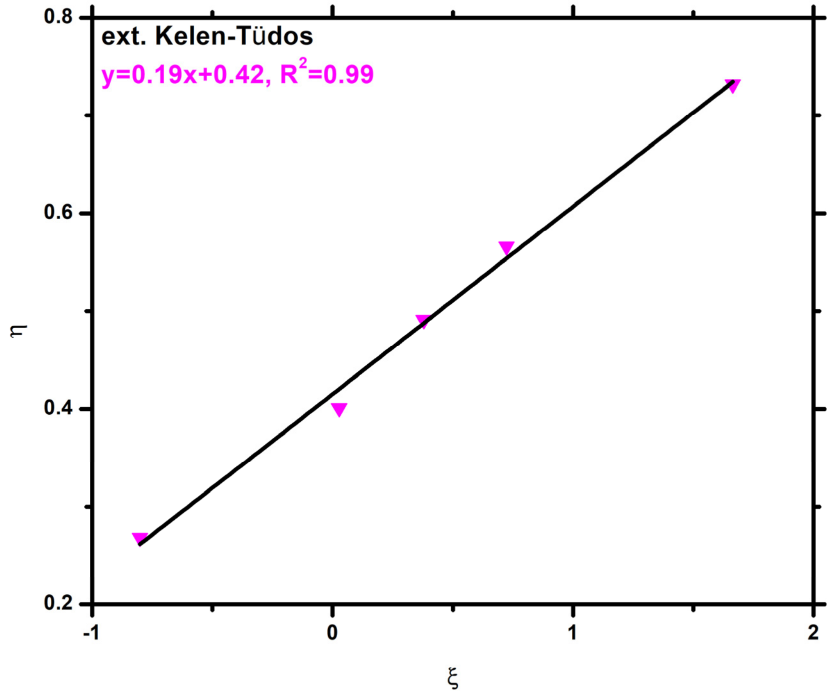

Figure S6. ext. K–T plot.

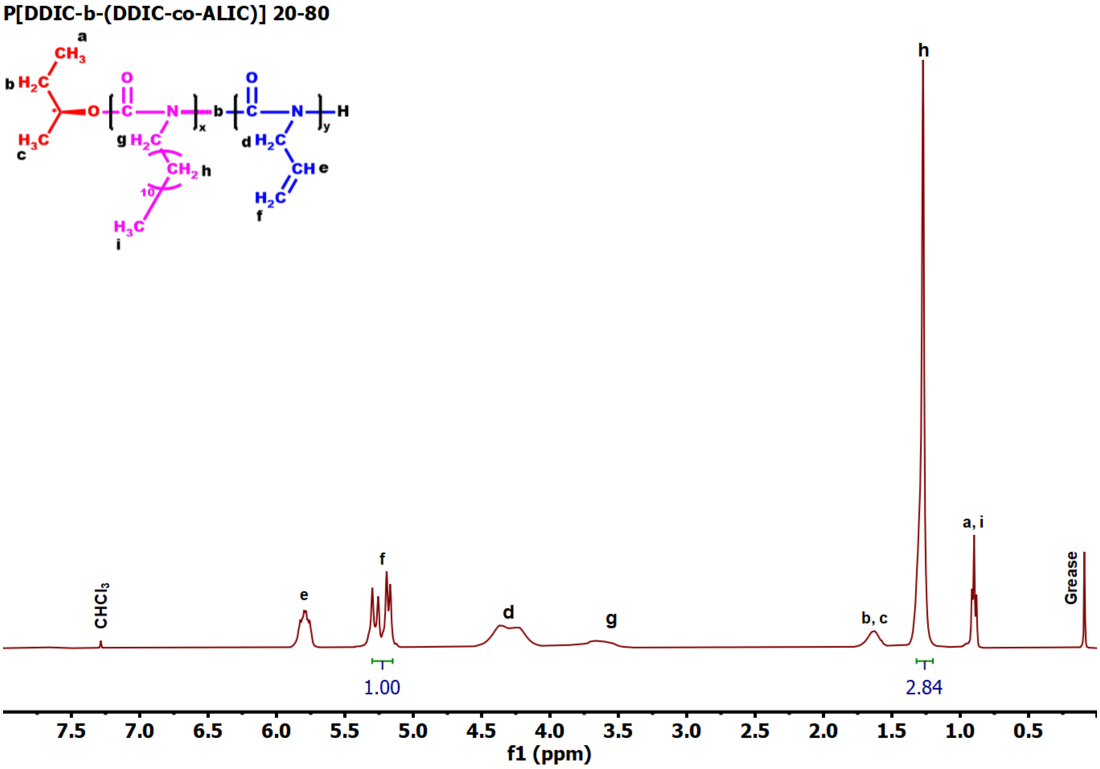

Figure S7. ^{1}H NMR spectrum of the sample P[DDIC–b–(DDIC-co-ALIC)] 20/80 in $CDCl_3$.

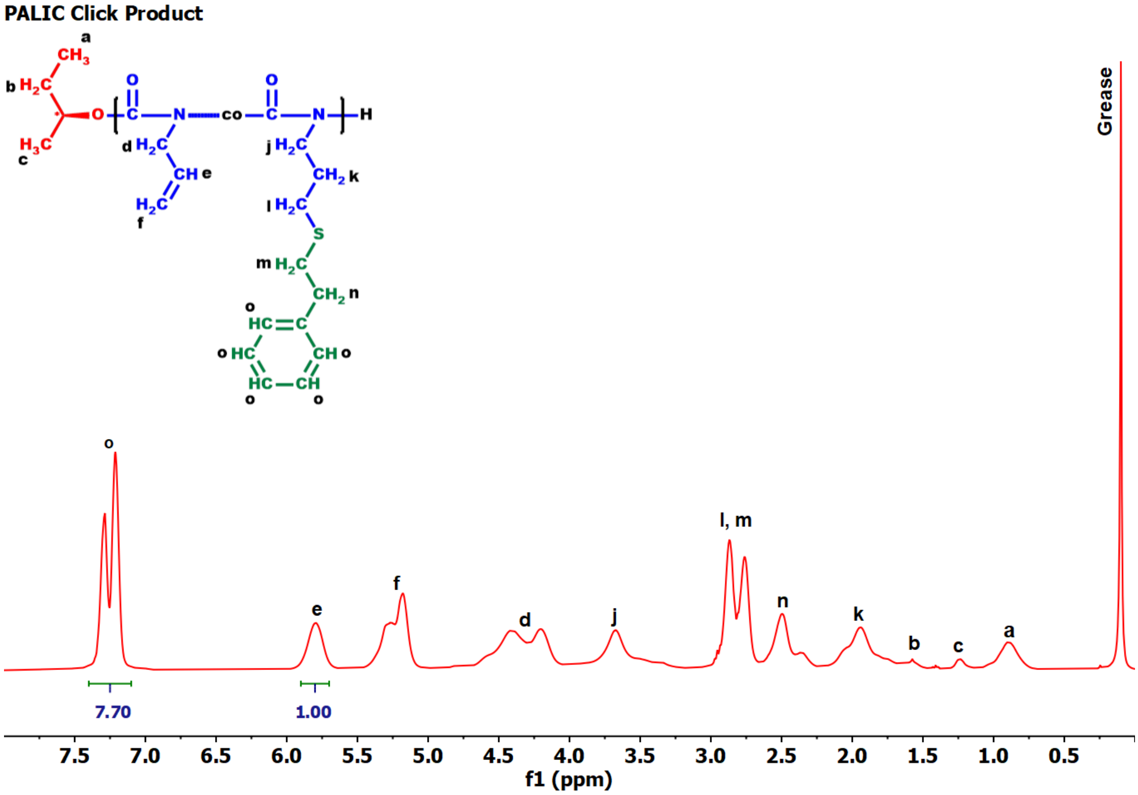

Figure S8. ^{1}H NMR spectrum of the sample PALIC Click product in $CDCl_3$.

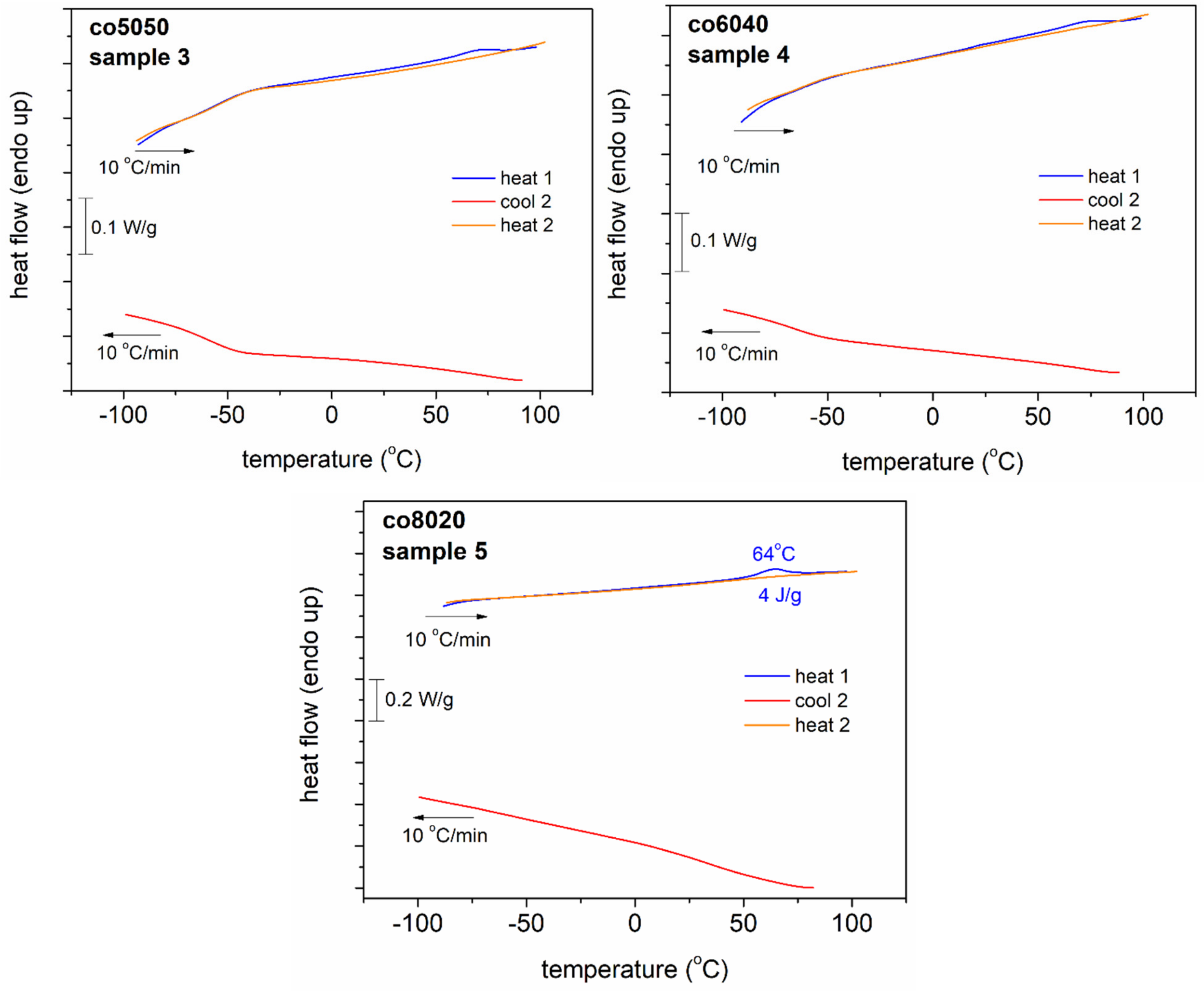

Figure S9. DSC traces for the statistical copolymers P(ALIC-co-DDIC) 50/50, 60/40, and 80/20.

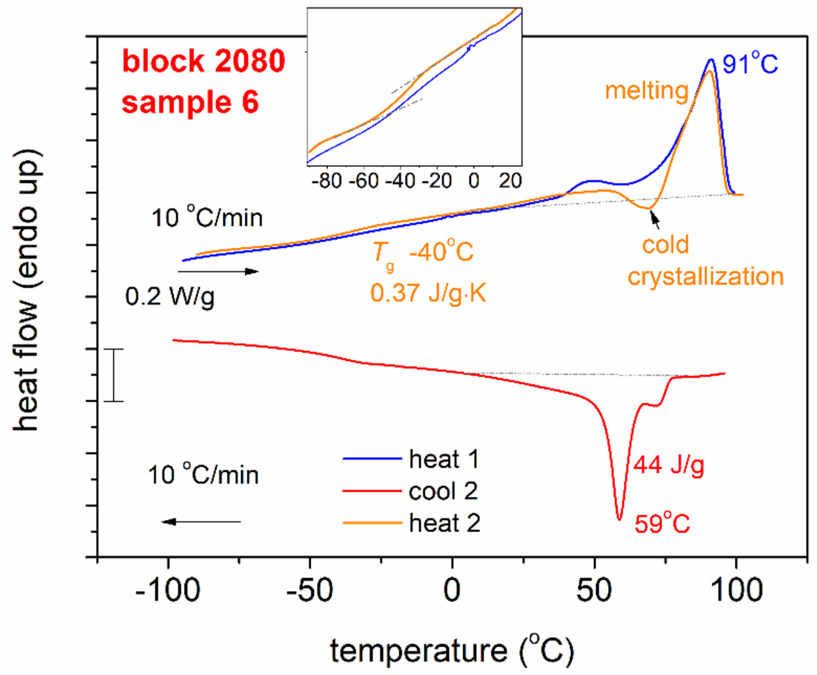

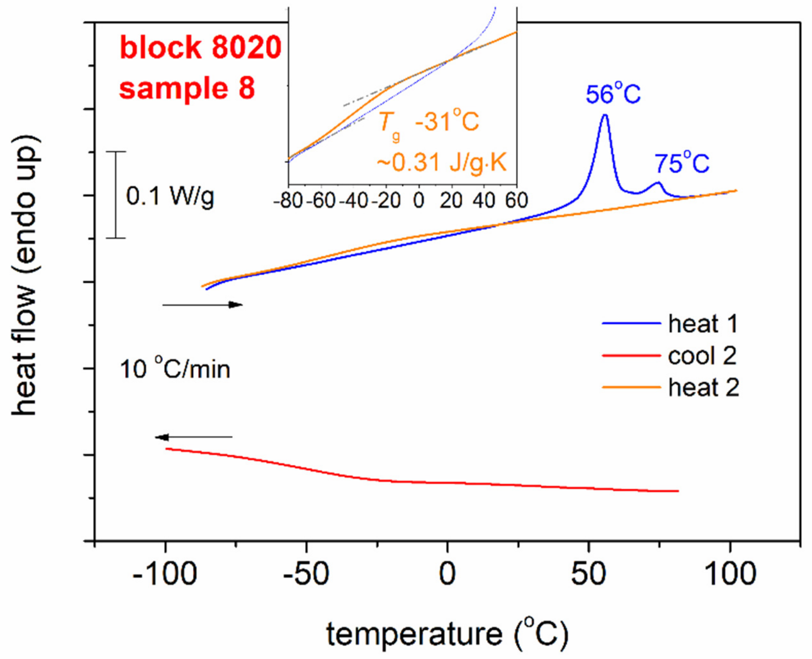

Figure S10. DSC traces of the block copolymers P[DDIC-b-(DDIC-co-ALIC)] 20/80 and 80/20 block copolymers.

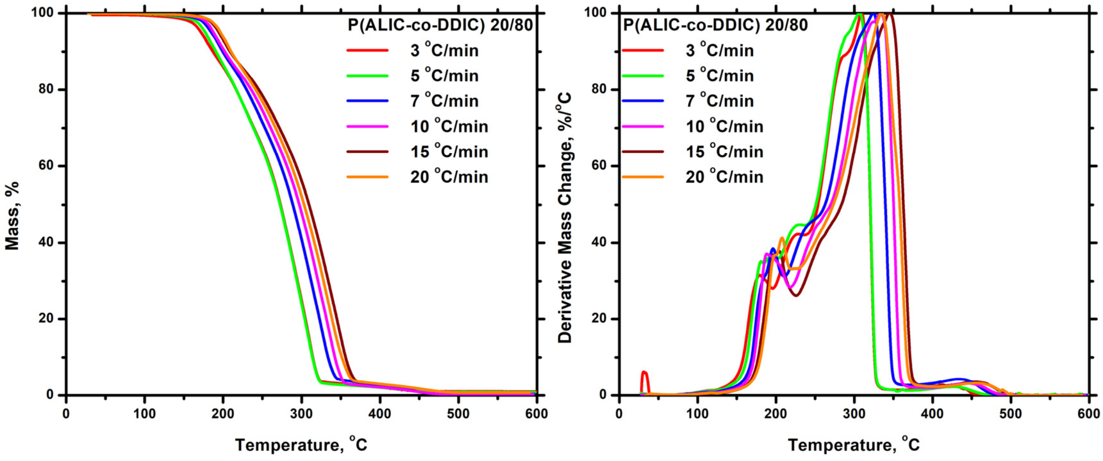

Figure S11. TGA (left) and DTG (right) plots for the P(ALIC–co–DDIC) 20/80 at all heating rates.

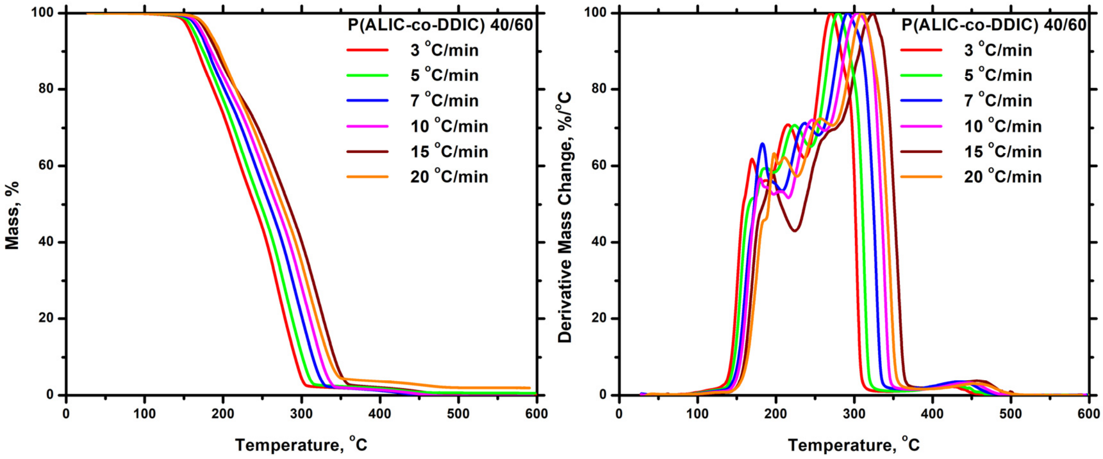

Figure S12. TGA (left) and DTG (right) plots for the P(ALIC–co–DDIC) 40/60 at all heating rates.

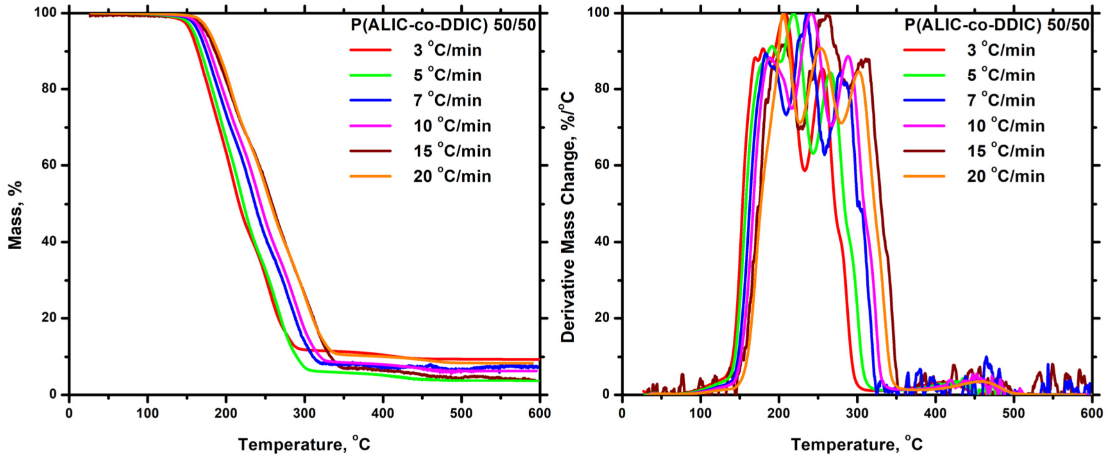

Figure S13. TGA (left) and DTG (right) plots for the P(ALIC–co–DDIC) 50/50 at all heating rates.

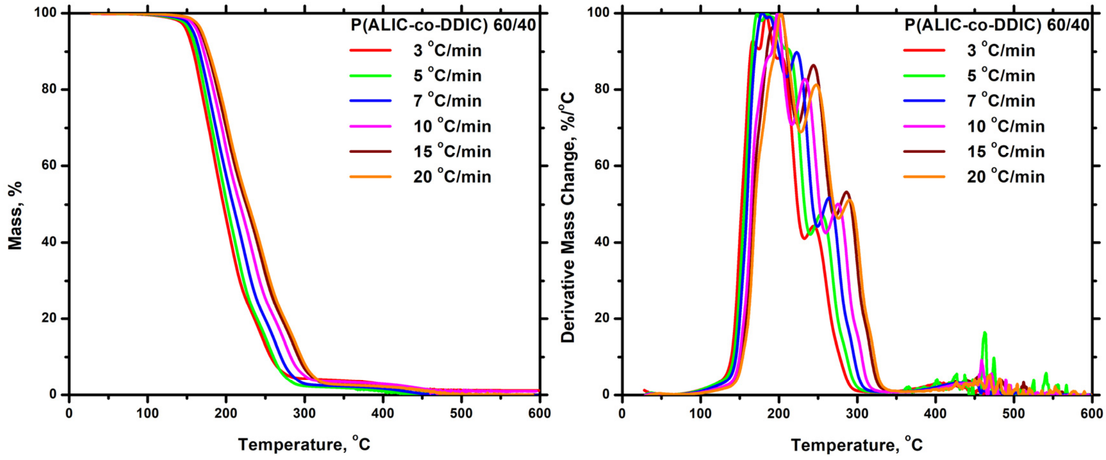

Figure S14. TGA (left) and DTG (right) plots for the P(ALIC–co–DDIC) 60/40 at all heating rates.

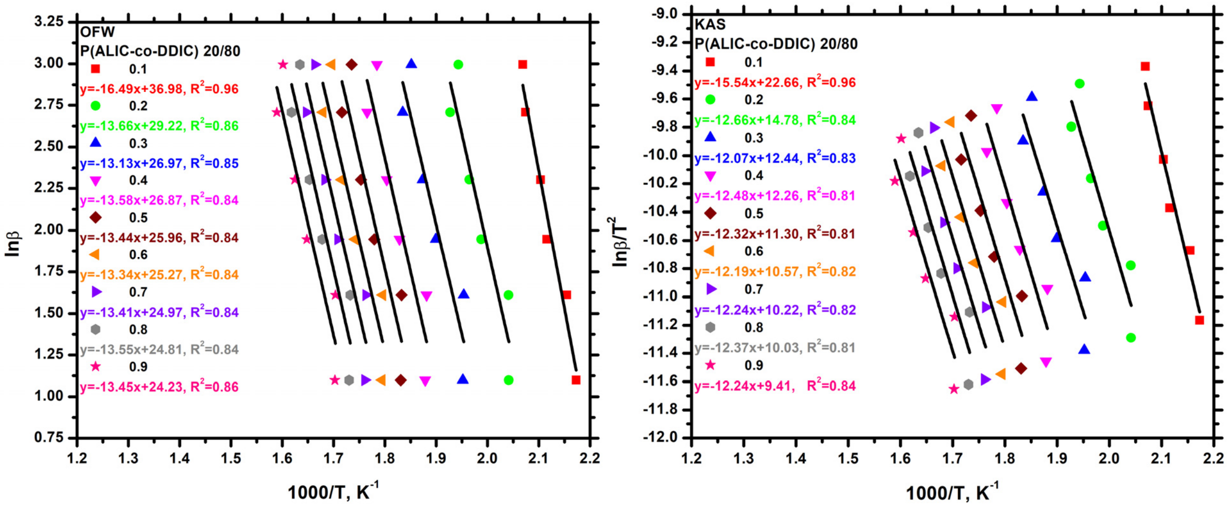

Figure S15. OFW and KAS plots for the P(ALIC-co-DDIC) 20/80 statistical copolymer.

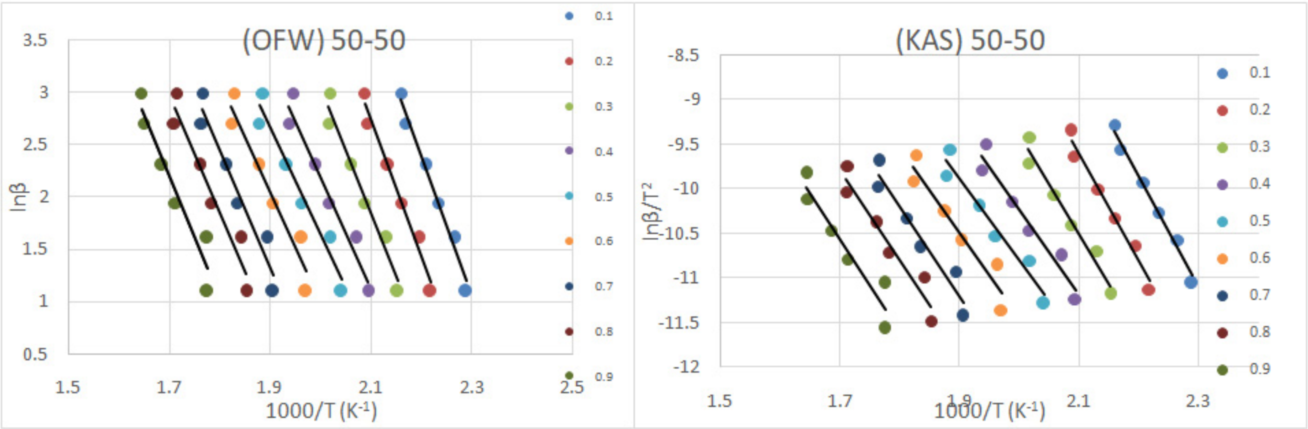

Figure S16. OFW and KAS plots for the P(ALIC–co–DDIC) 50-50 at all heating rates.

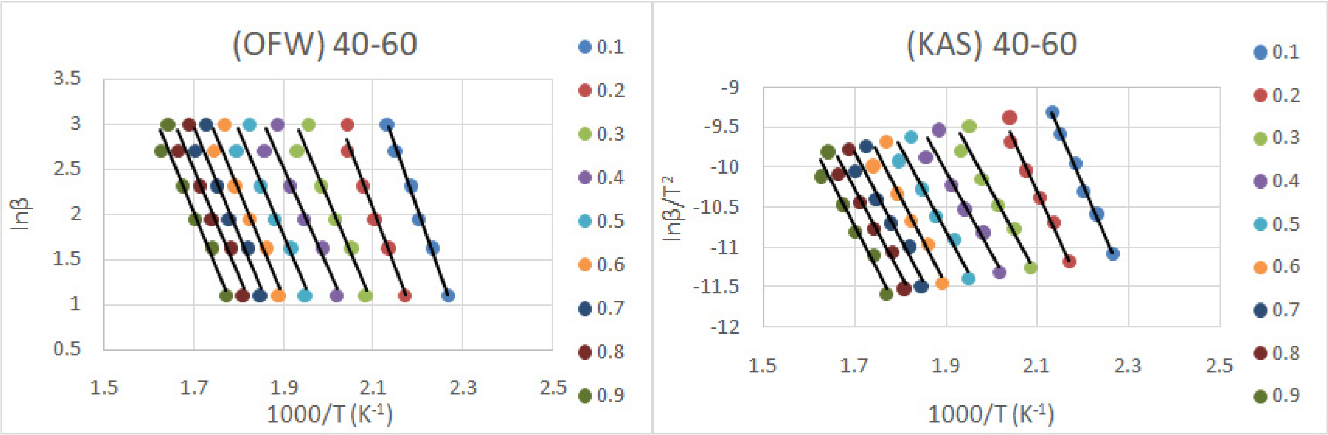

Figure S17. OFW and KAS plots for the P(ALIC–co–DDIC) 40-60 at all heating rates.

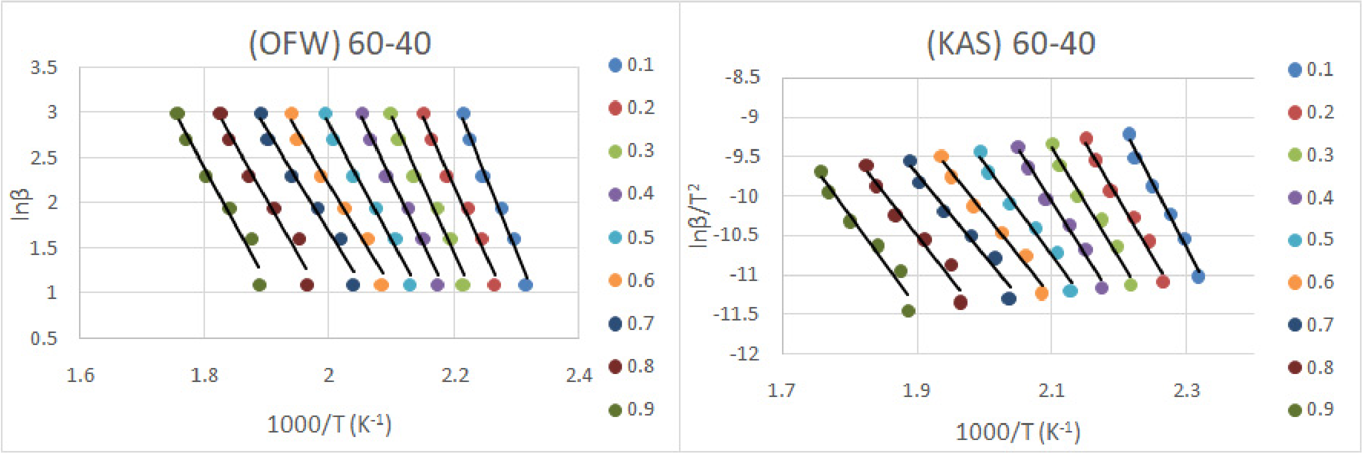

Figure S18. OFW and KAS plots for the P(ALIC–co–DDIC) 60-40 at all heating rates.

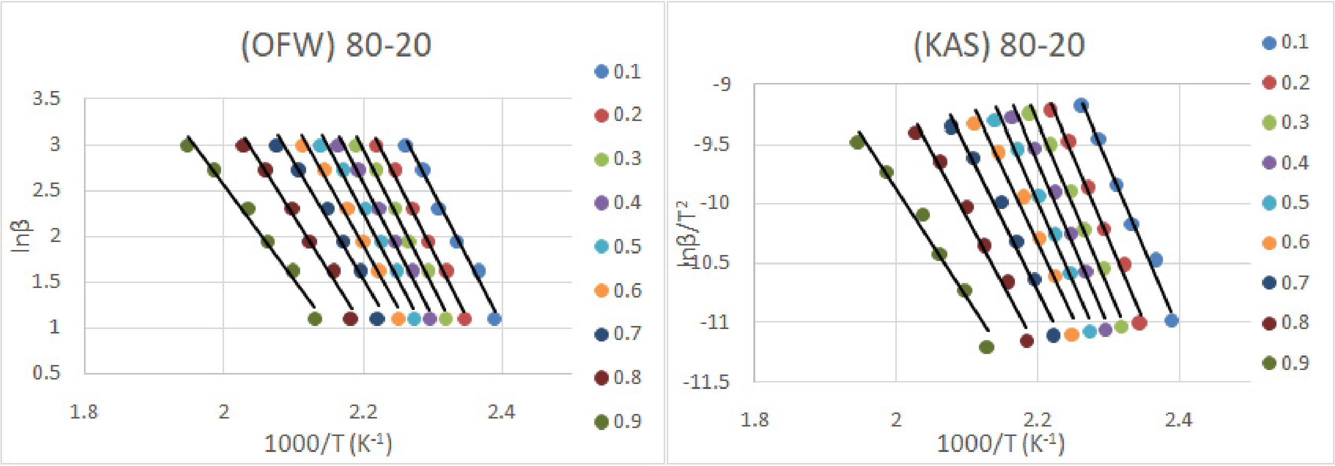

Figure S19. OFW and KAS plots for the P(ALIC–co–DDIC) 80-20 at all heating rates.

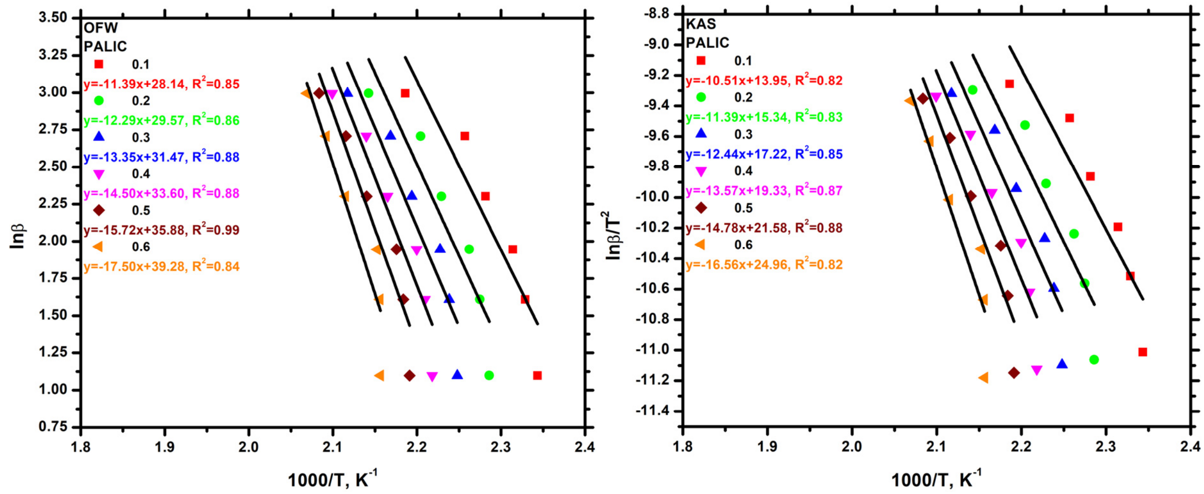

Figure S20. OFW (left) and KAS (right) plots for the PALIC.

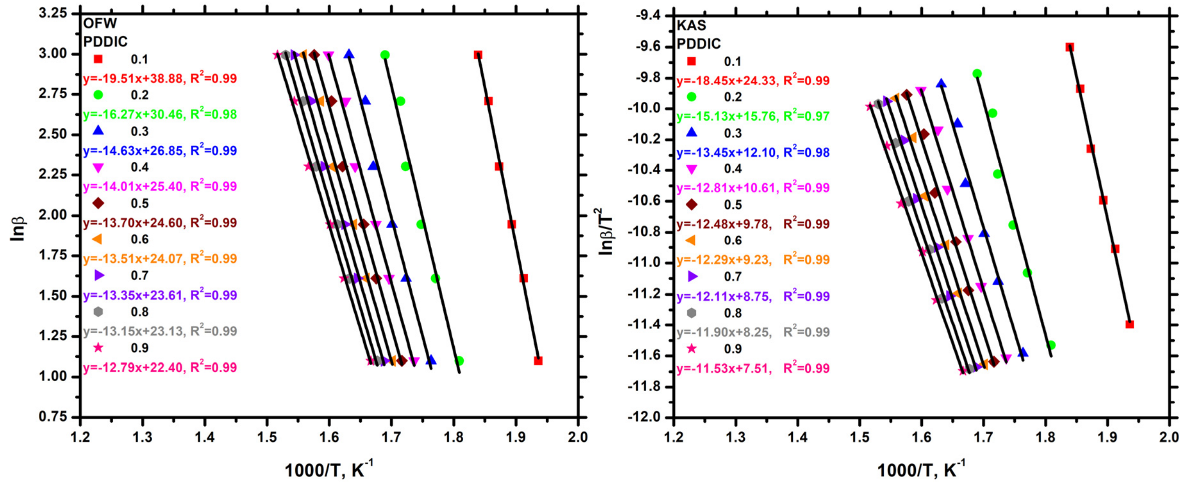

Figure S21. OFW (left) and KAS (right) plots for the PDDIC.

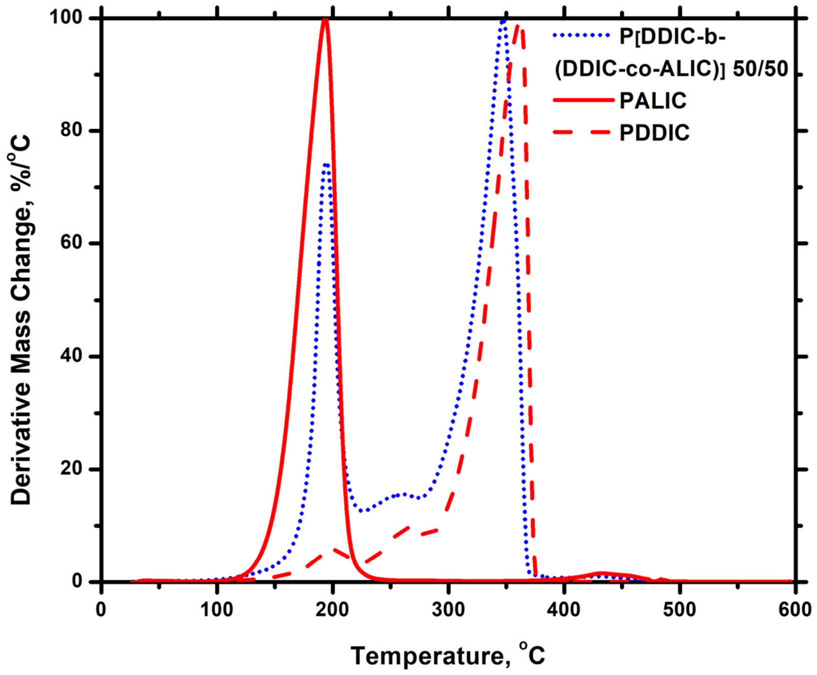

Figure S22. DTG plots for the PALIC and DDIC homopolymers and P[DDIC-b-(DDIC-co-ALIC)] 50/50 block copolymer at 10 °C.

Table S1. Parameters for the synthesis of the statistical copolymers P(ALIC-co-PDDIC).

ALIC/DDIC	Gm	Hm	Gm/Hm	1/Hm	η	ξ	η	Ξ
	F-R		inv F-R		K-T		ext K-T	
20/80	-0.5870	0.2092	-2.8052	4.7792	-0.7166	0.2554	-0.8008	0.2682
40/60	0.0261	0.4270	0.0612	2.3418	0.0252	0.4118	0.0276	0.4010
50/50	0.4127	0.5873	0.7027	1.7027	0.3447	0.4906	0.3792	0.4914
60/40	0.9452	0.8322	1.1358	1.2016	0.6554	0.5771	0.7234	0.5660
80/20	3.5556	1.7778	2.0000	0.5625	1.4891	0.7446	1.6642	0.7318

Table S2. Dyad sequences and mean sequence lengths for the statistical copolymers P(ALIC-co-PDDIC).

Sample	M(ALIC) - M(ALIC)	M(DDIC) - M(DDIC)	M(ALIC) - M(DDIC)	μ_{ALIC}	μ_{DDIC}
20/80	0.09147	0.63147	0.27707	1.66	5.56
40/60	0.32723	0.30723	0.36554	2.76	2.71
50/50	0.45703	0.19703	0.34595	3.64	2.14
60/40	0.57883	0.11883	0.30235	4.96	1.76
80/20	0.82217	0.02217	0.15566	11.56	1.29

Table S3. Molecular weights of the products of the click reactions.

PALIC			Click product			Yield of click reaction %
M_w	M_n	M_w/M_n	M_w	M_n	M_w/M_n	61
5940	4570	1.30	7590	6160	1.23	
P(ALIC-co-DDIC) 60-40			Click product			
M_w	M_n	M_w/M_n	M_w	M_n	M_w/M_n	89
17180	16340	1.05	22770	21080	1.08	

Table S4. Models for the thermal decomposition of PALIC.

PALIC			
MODEL	R^2	Ea	A
1. 1. Chemical reaction [$F_{1/3}$]	0.991	88.0029	1247304.34
1. 2. Chemical reaction [$F_{3/4}$]	0.995	95.6481	4221923.2
1. 3. Chemical reaction [$F_{3/2}$]	0.999	110.8554	637683179
1. 4. Chemical reaction [F_2]	0.999	121.991	3E+10
1. 5. Chemical reaction [F_3]	0.996	146.588	6E+13
1. 6. Chemical reaction [F_4]	0.989	173.85	2E+17
1. 7. Chemical reaction [G_1]	0.966	66.97	8573
1. 8. Chemical reaction [G_2]	0.936	54.447	325.2
1. 9. Chemical reaction [G_3]	0.898	44.284	20.754
2. 10. Nucleation (power law) [$P_{3/2}$]	0.987	127.143	4.1148E+10
2. 11. Nucleation (power law) [$P_{1/2}$]	0.983	37.33683	2.1838857
2. 12. Nucleation (power law) [$P_{1/3}$]	0.979	22.37052	0.031113
2. 13. Nucleation (power law) [$P_{1/4}$]	0.973	14.8832	0.0032
2. 14. Nucleation (parabolic law) [P_2]	0.988	172.017	4E+15
2. 15. Nucleation (exponential law) [E_1]	-	-	-
2. 16. Nucleation (exponential law) [E_2]	-	-	-
3. 17. Random nucleation/first order (Mampel) [A1,F1]	0.997	100.551	67375401.4
3. 18. Random nucleation (Arvami–Erofeev) [$A_{2/3}$]	0.997	154.566	9.604E+13
3. 19. Random nucleation (Arvami–Erofeev) [$A_{3/2}$]	0.997	64.51884	4582.8793
3. 20. Random nucleation (Arvami–Erofeev) [$A_{3/4}$]	0.997	136.616	9E+11
3. 21. Random nucleation (Arvami–Erofeev) [$A_{5/2}$]	0.996	35.6831	1.6666
3. 22. Random nucleation (Arvami–Erofeev) [A_2]	0.997	46.503	33.91
3. 23. Random nucleation (Arvami–Erofeev) [A_3]	0.996	28.478	0.213
3. 24. Random nucleation (Arvami–Erofeev) [A_4]	0.995	19.462	0.015
3. 25. Random nucleation (Arvami–Erofeev) [$A_{1/2}$]	0.997	208.664	1E+20
3. 26. Random nucleation (Arvami–Erofeev) [$A_{1/3}$]	0.997	316.777	1.6E+32
3. 27. Random nucleation (Arvami–Erofeev) [$A_{1/4}$]	0.997	424.89	2E+44
3. 28. Branching nuclei (Prout–Tompkins) [B_1]	0.338	-427.9	0
4. 29. Contracting disk [R_1,F_0,P_1]	0.986	82.24407	357473.593
4. 30. Contracting cylinder [R_2,$F_{1/2}$]	0.993	90.9945	2215357.9
4. 31. Contracting sphere [R_3,$F_{2/3}$]	0.995	94.0692	3573857.7
5. 32. One-dimensional diffusion [D_1]	0.988	172.017	4.1121E+15
5. 33. Three-dimensional diffusion [D_2]	0.992	183.2355	4.829E+16
5. 34. Three-dimensional diffusion (Jander) [D_3]	0.995	195.7836	3.59E+17
5. 35. Three-dimensional diffusion (Ginstling–Brounshtein) [D_4]	0.993	187.391	3E+16
5. 36. Three-dimensional diffusion (Crank) [D_5]	0.999	222.293	6E+20
5. 37. Three-dimensional diffusion [D_6]	0.978	145.92	3E+11
5. 38. Three-dimensional diffusion [D_7]	0.985	162.96	3E+13
5. 39. Three-dimensional diffusion [D_8]	0.978	145.92	3E+11
6. 40. [G_7]	0.991	41.72451	5.77890495
6. 41. [G_8]	0.993	43.27017	7.4901511

Table S5. Models for the thermal decomposition of PDDIC.

PDDIC			
**MODEL**	R^2	Ea	A
**1. 1. Chemical reaction [$F_{1/3}$]**	0.922	57.03984	4.91295432
**1. 2. Chemical reaction [$F_{3/4}$]**	0.891	65.37477	13.056184
**1. 3. Chemical reaction [$F_{3/2}$]**	0.825	83.5155	1737.4221
**1. 4. Chemical reaction [F_2]**	0.78	97.8087	90594
**1. 5. Chemical reaction [F_3]**	0.705	130.799	3E+08
**1. 6. Chemical reaction [F_4]**	0.655	167.86	2E+12
**1. 7. Chemical reaction [G_1]**	0.977	37.52	0.131
**1. 8. Chemical reaction [G_2]**	0.980	27.938	0.017
**1. 9. Chemical reaction [G_3]**	0.961	20.9828	0.0035
**2. 10. Nucleation (power law) [$P_{3/2}$]**	0.948	81.67068	808.208886
**2. 11. Nucleation (power law) [$P_{1/2}$]**	0.918	20.75838	0.0027205
**2. 12. Nucleation (power law) [$P_{1/3}$]**	0.874	10.60356	0.0002153
**2. 13. Nucleation (power law) [$P_{1/4}$]**	0.778	5.52615	4E-05
**2. 14. Nucleation (parabolic law) [P_2]**	0.951	112.102	305142
**2. 15. Nucleation (exponential law) [E_1]**	-	-	-
**2. 16. Nucleation (exponential law) [E_2]**	-	-	-
**3. 17. Random nucleation/first order (Mampel) [A1,F1]**	0.870	70.97571	191.993821
**3. 18. Random nucleation (Arvami–Erofeev) [$A_{2/3}$]**	0.879	111.2709	717187.97
**3. 19. Random nucleation (Arvami–Erofeev) [$A_{3/2}$]**	0.855	44.08455	0.6691379
**3. 20. Random nucleation (Arvami–Erofeev) [$A_{3/4}$]**	0.877	97.8087	47200
**3. 21. Random nucleation (Arvami–Erofeev) [$A_{5/2}$]**	0.814	22.57	0.0054
**3. 22. Random nucleation (Arvami–Erofeev) [A_2]**	0.836	30.639	0.035
**3. 23. Random nucleation (Arvami–Erofeev) [A_3]**	0.786	17.193	0.001
**3. 24. Random nucleation (Arvami–Erofeev) [A_4]**	0.712	10.471	2E-04
**3. 25. Random nucleation (Arvami–Erofeev) [$A_{1/2}$]**	0.883	151.658	2E+09
**3. 26. Random nucleation (Arvami–Erofeev) [$A_{1/3}$]**	0.887	232.265	2E+16
**3. 27. Random nucleation (Arvami–Erofeev) [$A_{1/4}$]**	0.889	312.955	2E+23
**3. 28. Branching nuclei (Prout–Tompkins) [B_1]**	0.425	-384.3	-0
**4. 29. Contracting disk [R_1,F_0,P_1]**	0.943	51.21453	1.84623562
**4. 30. Contracting cylinder [R_2,$F_{1/2}$]**	0.911	60.22257	7.8159982
**4. 31. Contracting sphere [R_3,$F_{2/3}$]**	0.898	63.60474	11.528399
**5. 32. One-dimensional diffusion [D_1]**	0.951	112.1019	305142.379
**5. 33. Three-dimensional diffusion [D_2]**	0.934	123.1542	1896579.8
**5. 34. Three-dimensional diffusion (Jander) [D_3]**	0.910	136.8657	9380331.3
**5. 35. Three-dimensional diffusion (Ginstling–Brounshtein) [D_4]**	0.926	127.642	1E+06
**5. 36. Three-dimensional diffusion (Crank) [D_5]**	0.854	167.945	1E+10
**5. 37. Three-dimensional diffusion [D_6]**	0.969	91.742	285.7
**5. 38. Three-dimensional diffusion [D_7]**	0.957	104.96	6366
**5. 39. Three-dimensional diffusion [D_8]**	0.969	91.742	285.7
**6. 40. [G_7]**	0.881	25.2624	0.0062852
**6. 41. [G_8]**	0.866	26.95764	0.007926

References

S1. Fineman, M.; Ross, S.D. Linear method for determining monomer reactivity ratios in copolymerization. *J. Polym. Sci.* **1950**, *5*, 259–262.

S2. Kelen, T.; Tüdos, F. Analysis of the Linear Methods for Determining Copolymerization Reactivity Ratios. I. A New Improved Linear Graphic Method. *J. Macromol. Sci. Part A Chem.* **1975**, *9*, 1–27.
S3. Igarashi, S. Representation of composition and blockiness of the copolymer by a triangular coordinate system. *J. Polym. Sci. Part B Polym. Lett.* **1963**, *1*, 359–363.
S4. Elias, H.G. *Synthesis, Materials, and Technology*; Springer: New York, NY, USA, 1984; Volme 2.
S5. Galukhin, A.; Liavitskaya, T.; Vyazovkin, S. Kinetic and mechanistic insights into thermally initiated polymerization of cyanate esters with different bridging groups. *Macromol. Chem. Phys*. **2019**, *220*, 1900141.
S6. Boulkadid, M.K.; Touidjine, S.; Trache, D.; Belkhiri, S. Analytical methods for the assessment of curing kinetics of polyurethane binders for high energy composites. *Crit. Rev. Anal. Chem*. **2022**, *52*, 1112–1121.
S7. Tarchoun, A.F.; Trache, D.; Klapötke, T.M.; Chelouche, S.; Derradji, M.; Bessa, W.; Mezroua, A. A promising energetic polymer from Posidonia oceanica brown alge: Synthesis, characterization, and kinetic modeling. Macromol. *Chem. Phys*. **2019**, *220*, 1900358.
S8. Liqing, L.; Donghua, C. Application of iso-temperature method of multiple rate to kinetic analysis. Dehydration for calcium oxalate monohydrate. *J. Therm. Anal. Calorim.* **2004**, *78*, 283–293.
S9. Lim, A.C.R.; Chin, B.L.F.; Jawad, Z.A.; Hii, K.L. Kinetic analysis of rice husk pyrolysis using Kissinger-Akahira-Sunose (KAS) method. *Procedia Eng.* **2016**, *148*, 1247–1251.
S10. Hayoune, F.; Chelouche, S.; Trache, D.; Zitouni, S.; Grohens, Y. Thermal decomposition kinetics and lifetime prediction of a PP/PLA blend supplemented with iron stearate during artificial aging. Thermochim. Acta **2020**, *690*, 178700.
S11. Trache, D.; Abdelaziz, A.; Siouani, B. A simple and linear isoconversional method to determine the pre-exponential factors and the mathematical reaction mechanism functions. *J. Therm. Anal. Calorim*. **2017**, *128*, 335–348.
S12. Ozawa, T. A new method of analyzing thermogravimetric data. *Bull. Chem. Soc. Jpn*. **1965**, *38*, 1881–1886.
S13. Flynn, J.H.; Wall, L.A. A quick, direct method for the determination of activation energy from thermogravimetric data. *J. Polym. Sci. Polym. Lett. Ed.* **1966**, *4*, 323–328.
S14. Ozawa, T. Kinetic analysis of derivative curves in thermal analysis. *J. Therm. Anal. Calorim.* ***1970***, *2*, 301–324.
S15. Kissinger, H.E. Reaction Kinetics in Differential Thermal Analysis. *Anal. Chem.* **1957**, *29*, 1702–1706.

Disclaimer/Publisher's Note: The statements, opinions and data contained in all publications are solely those of the individual author(s) and contributor(s) and not of MDPI and/or the editor(s). MDPI and/or the editor(s) disclaim responsibility for any injury to people or property resulting from any ideas, methods, instructions or products referred to in the content.
